# A high-throughput method to deliver targeted optogenetic stimulation to moving *C*. *elegans* populations

**DOI:** 10.1371/journal.pbio.3001524

**Published:** 2022-01-28

**Authors:** Mochi Liu, Sandeep Kumar, Anuj K. Sharma, Andrew M. Leifer

**Affiliations:** 1 Department of Physics, Princeton University, Princeton, New Jersey, United States of America; 2 Princeton Neuroscience Institute, Princeton University, Princeton, New Jersey, United States of America; Brandeis University, UNITED STATES

## Abstract

We present a high-throughput optogenetic illumination system capable of simultaneous closed-loop light delivery to specified targets in populations of moving *Caenorhabditis elegans*. The instrument addresses three technical challenges: It delivers targeted illumination to specified regions of the animal’s body such as its head or tail; it automatically delivers stimuli triggered upon the animal’s behavior; and it achieves high throughput by targeting many animals simultaneously. The instrument was used to optogenetically probe the animal’s behavioral response to competing mechanosensory stimuli in the the anterior and posterior gentle touch receptor neurons. Responses to more than 43,418 stimulus events from a range of anterior–posterior intensity combinations were measured. The animal’s probability of sprinting forward in response to a mechanosensory stimulus depended on both the anterior and posterior stimulation intensity, while the probability of reversing depended primarily on the anterior stimulation intensity. We also probed the animal’s response to mechanosensory stimulation during the onset of turning, a relatively rare behavioral event, by delivering stimuli automatically when the animal began to turn. Using this closed-loop approach, over 9,700 stimulus events were delivered during turning onset at a rate of 9.2 events per worm hour, a greater than 25-fold increase in throughput compared to previous investigations. These measurements validate with greater statistical power previous findings that turning acts to gate mechanosensory evoked reversals. Compared to previous approaches, the current system offers targeted optogenetic stimulation to specific body regions or behaviors with many fold increases in throughput to better constrain quantitative models of sensorimotor processing.

## Abstract 2

This study resents a new targeted illumination method for the nematode Caenorhabditis elegans, allowing delivery of optogenetic stimulation to specific body parts of many animals at once, automatically triggered by the animals’ behavior.

## Introduction

How sensory signals are transformed into motor outputs is a fundamental question in systems neuroscience [1]. Optogenetics [2,3], coupled with automated measures of behavior [4,5], has emerged as a useful tool for probing sensorimotor processing, especially in small model organisms [6]. In optically transparent animals, such as *Caenorhabditis elegans* and *Drosophila*, such optogenetic manipulations can be performed noninvasively by illuminating an animal expressing light-gated ion channels [7]. Optogenetically perturbing neural activity and observing behavior has been widely used to study specific neural circuits, such as those involved in chemotaxis [8–11] (reviewed for *Drosophila* in [12]), olfaction [13], learning and memory [14,15], and locomotion and escape [16–19], to name just a few examples. In *Drosophila*, high-throughput optogenetic delivery to behaving animals has been used to screen libraries of neural cell types and map out previously unknown relations between neurons and behavior [20].

Optogenetic investigations of neural circuits underlying behavior confront three technical challenges: the first is to deliver stimulation targeted only to the desired neuron or neurons; the second is to deliver the stimulus at the correct time in order to probe the circuit in a relevant state or behavioral context; and the third is to efficiently acquire enough observations of stimulus and response to draw meaningful conclusions. Existing methods each address some of these challenges, but not all three.

To stimulate only desired cells, the expression of optogenetic proteins is typically restricted to specific cells or cell types [21–23]. If cell-specific genetic drivers are not readily available, then genetic specificity can be complemented with optical targeting. Patterned light from a projector, for example, can be used to illuminate only a subset of the cells expressing the optogenetic protein [24,25]. For targeting behaving animals, real-time processing is also needed to track the animal and dynamically update the illumination pattern based on its movements [26–31].

Closed-loop approaches are further needed to deliver a perturbation timed to a specific behavior. For example, delivering a perturbation triggered to the swing of an animal’s head has informed our understanding of neural mechanisms underlying thermotaxis [32] and chemotaxis [8]. The use of closed-loop stimulation triggered on behavior in *C*. *elegans* [31,33], *Drosophila* [34], and mammals [35,36] is part of a broader trend in systems neuroscience toward more efficient exploration of the vast space of possible neural perturbations [37,38], especially during ethologically relevant naturalistic behaviors [39].

Targeted and closed-loop illumination systems probe one animal at a time [8,26,31,40,41] or, at most, two [42]. This low throughput poses challenges for acquiring datasets with enough statistical power to constrain quantitative models of neural computation. To increase throughput, a separate body of work simultaneously measures behavior of populations of many animals in an arena, in order to amass thousands of animal hours of recordings [43–47]. Delivering spatially uniform optogenetic perturbations to such populations has helped constrain quantitative models of sensorimotor processing of chemotaxis and mechanosensation [9,10,48]. Because the entire body of every animal is illuminated, this approach relies entirely on genetics for targeting. Recent work has used stochastic spatially varying illumination patterns in open loop from a projector [49] or cellphone [50] to probe the spatial dependence of optogenetic stimulation. But because these methods are open loop, they cannot target stimulation specifically to any animal or body part. Instead, they rely on after the fact inspection of where their stimuli landed, decreasing throughput.

A recent system for zebrafish demonstrated the potential of closed-loop illumination in a multianimal setting [51]. That work probed the zebrafish optomotor response in high throughput by presenting patterned closed-loop visual stimuli, such as moving gratings, to many different animals simultaneously. That work also had an optogenetic component, but it relied on a low-resolution LED array that was restricted to open-loop full-field illumination and therefore could not target regions within animals or even target individual animals.

Here, we demonstrate a closed-loop real-time targeting system for *C*. *elegans* that tackles all three challenges: targeting, closed-loop triggering, and throughput. The system delivers targeted illumination to specified parts of the animal’s body, stimulus delivery can be be triggered automatically on behavior, and the system achieves a high throughput by tracking and independently delivering targeted stimuli to populations of animals simultaneously. We apply this system to the *C*. *elegans* mechanosensory circuit [52,53] to characterize how competing stimuli in anterior and posterior mechanosensory neurons are integrated by downstream circuity. We also revisit our prior observation that turning behavior alters the animals, likelihood of responding to mechanosensory stimuli [48]. We deliver closed-loop stimulation triggered to the onset of turning to obtain a dataset with two orders of magnitude more stimulus events compared to that investigation. We use these measurements to validate our initial observation with greater statistical power.

## Results

**Fig 1:**
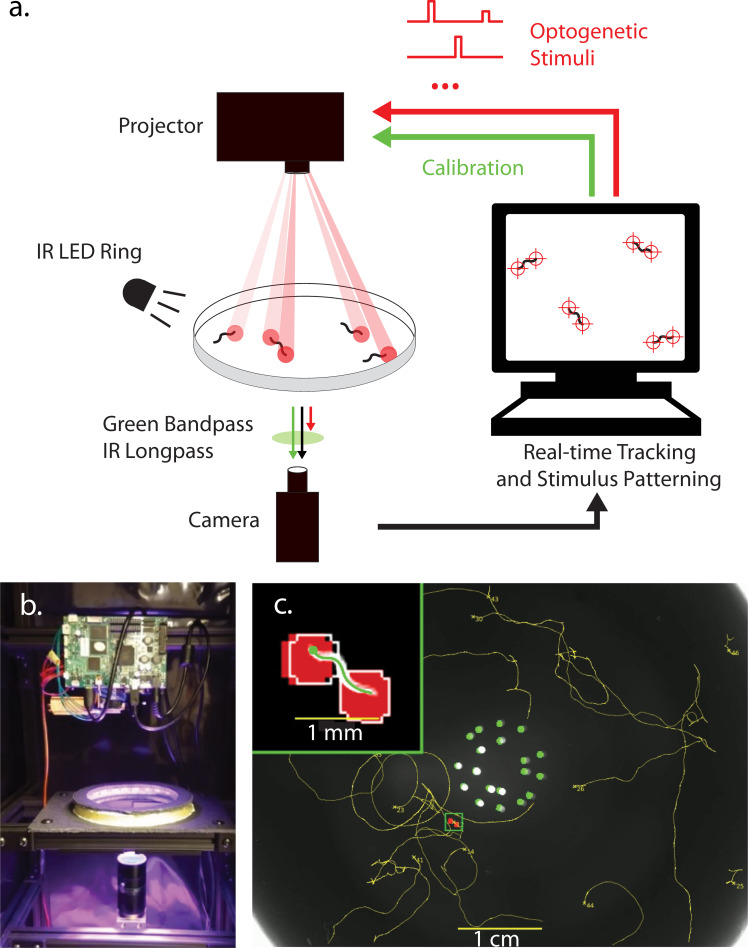
Closed-loop targeted optogenetic delivery system. **(a)** Schematic of system. A projector simultaneously delivers patterned targeted optogenetic stimulation to multiple worms on an agar plate in real time. **(b)** Photograph of instrument. **(c)** Image from experiment shows animals expressing Chrimson in touch receptor neurons (AML470) as they crawl on agar. Corresponding video is shown in S1 Video. Tracked paths are shown in yellow. Green and white dots in the center relate to a visual time stamping system and are excluded from analysis. Inset shows details of an animal receiving optogenetic stimulation targeted to its head and tail (0.5-mm diameter stimuli). The two white circle in the inset show the targeted location of the stimulus. Red shading shows area where stimulus was delivered.

We developed a closed-loop targeted optical delivery system to illuminate specified regions, such as the head or tail, in populations of *C*. *elegans* expressing the optogenetic protein Chrimson in the six gentle touch receptor neurons (AVM, ALML, ALMR, PVM, PLML, and PLMR) under a *mec-4* promoter as the animals crawled on agar in a 9-cm dish. The system used red light (peak 630 nm) from a custom color projector made of an optical engine (Anhua M5NP) driven by a separate control board, described in Materials and methods. Animal behavior was simultaneously recorded from a CMOS camera (Fig 1A and 1B). Dark field illumination from a ring of infrared LEDs (peak emission at 850 nm) was used to observe animal behavior because this avoided exciting Chrimson. Optical filters allowed the infrared illuminated behavior images to reach the camera, but blocked red or blue light coming from the projector. Green light from the projector was used to present visual timestamps and other spatiotemoporal calibration information to the camera. Custom real-time computer vision software monitored the pose and behavior of each animal and generated patterned illumination restricted to only specified regions of the animal, such as its head or tail, optionally triggered by the animal’s behavior (Fig 1C, S1 Video).

### Anterior and posterior mechanosensory response

The mechanosensory response to anterior or posterior stimulation has been probed extensively by softly touching the animal with a hair [52,54,55], tapping the substrate on which the animal crawls [52,56], automatically applying forces via microelectromechanical force probes [57–59] or applying forces via a microfluidic device [60]. These approaches have identified dozens of genes related to touch sensation [61].

Mechanosensory responses have also been probed optogenetically by expressing light-gated ion channels in the six gentle touch mechanosenory neurons ALML, ALMR, AVM, PVM, PLML, and PLMR [7]. Targeted illumination experiments showed that optogenetically stimulating posterior touch neurons PLML and PLMR was sufficient to elicit the animal to speed up or sprint and that stimulating anterior gentle touch neurons (ALML and ALMR) or AVM was sufficient to induce the animal to move backward in a reversal behavior [26,27,29,30]. Those experiments were conducted one worm at a time, to accommodate the patterned illumination systems that were needed to restrict illumination to only a portion of the animal. Here, we revisit these experiments, this time by probing a population of animals simultaneously to achieve higher sample sizes.

**Fig 2:**
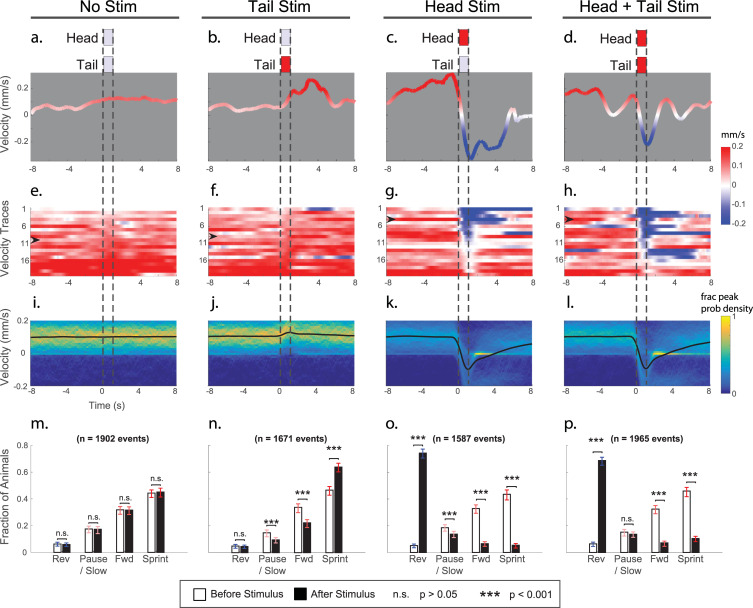
Stimulation of anterior mechanosensory neurons evokes reverse behavior; stimulation of posterior mechanosensory neurons evokes sprints. Targeted stimulation is delivered to worms expressing Chrimson in their gentle touch mechanosensory neurons (strain AML470). **(a–d)** Representative velocity trace from single stimulus event. Dashed lines indicate timing of stimulation (1 second, 0.5-mm diameter circular illumination pattern centered at the tip of the animal’s head and/or tail, red light intensity of 80 uW/mm^2^). **(e–h)** A total of 20 randomly sampled velocity traces for each stimulus condition are shown, sorted by mean velocity immediately after stimulation. Same as in S2–S5 Videos. Arrow indicates each representative trace shown in **a–d**. **(i–l)** Probability density of velocities for each stimulus condition. Mean velocity is shown as black line. *n*>1,500 stimulus events per condition. **(m–p)** The fraction of animals occupying each of 4 velocity-defined behavioral states is shown for the time point 2 seconds before stimulation onset and immediately after stimulation. Cutoffs for behavior states are shown in S3 Fig. *p*-Values calculated using Wilcoxon rank sum test. Error bars represents 95% confidence intervals estimated using 1,000 bootstraps. Numerical values are listed in S1 Data.

We delivered 1 second of red light illumination to the head, tail, or both of transgenic animals expressing the light-gated ion channel Chrimson in the 6 gentle touch mechanosensory neurons (500-um diameter stimulus region, 30-second interstimulus interval). Multiple combinations of illumination intensities were used, but, here, only the 80 uW/mm^2^ intensity stimuli are considered (Fig 2). The system independently tracked or stimulated an average of 12 worms on a plate simultaneously (S1 Table, distribution shown in S1F Fig). Across four days, 95 plates were imaged using four identical instruments running in parallel, for a total of 74,693 stimulation events across all stimulus intensity combinations. A total of 43,418 stimulus events met our inclusion criteria for valid worm body shape and track length. Of those, there were 7,125 stimulus events with an illumination intensity of 80 uW/mm^2^, at least 1,500 stimulus events of each of the following conditions: head illumination, tail illumination, both, or neither. For comparison, Leifer and colleagues [26] used 14 stimulus events, a two-order of magnitude difference in sample size.

Consistent with prior reports, activating posterior mechanosensory neurons by delivering light to the tail resulted in an increase in sprints and an overall increase in average velocity (Fig 2, S3 Video). Similarly, activating anterior mechanosensory neurons by delivering light to the head resulted in an increase in reversal events and a decrease in average velocity (S4 Video). Simultaneous stimulation to head and tail resulted in an increase in reversals (S5 Video). Animals were classified as performing forward locomotion, pause/slow, sprint or reversals, based on their velocity (S3 Fig). The animal showed little response to control conditions in which no light was delivered (Fig 2A, 2E, 2I, and 2M, S2 Video) or in which the necessary cofactor all-trans-retinal (ATR) was withheld (S4 Fig).

We further stimulated a different strain of animals that also express Chrimson in the gentle touch neurons and that we had used previously, AML67 [48]. That strain had a slighlty different genetic background and had been constructed with a higher DNA plasmid concentration during microinjection (40 ng/ul for AML67, compared to 10 ng/ul for AML470 used above). The AML67 strain behaved largely the same on this instrument in response to simultaneous anterior and posterior stimulation as it had in [48], although with slightly reduced effect size (S6 Fig). With this instrument’s ability to probe anterior and posterior responses separately, we further revealed other minor differences between the two strains. For example, tail stimulation to AML67 caused a very slight decrease in sprints. By contrast, tail stimulation to AML470 evoked an increase, which is what we would have expected from the literature. One possible explanation is that AML67 has less light sensitivity, perhaps due to silencing or due to defects in trafficking resulting from Chrimson over expression.

### Integration of conflicting anterior and posterior mechanosensory signals

Mechanosensory neurons act as inputs to downstream interneurons and motor neurons that translate mechanosensory signals into a behavioral response [19,53,56,62,63]. A growing body of evidence suggests that downstream circuitry relies on the magnitude of signals in both the anterior and posterior mechanosensory neurons to determine the behavioral response. For example, a plate tap activates both anterior and posterior mechanosensory neurons and usually elicits a reversal response [45,48,54,56]. But the animal’s response to tap can be biased toward one response or another by selectively killing specific touch neurons via laser ablation [56]. For example, if ALMR alone is ablated, the animal is more balanced in its response and is just as likely to respond to a tap by reversing as it is by sprinting. If both ALML and ALMR are ablated, the animal will then sprint the majority of the time [56]. Competing anterior–posterior optogenetic stimulation of the mechanosensory neurons also influences the animal’s behavioral response. For example, a higher intensity optogenetic stimulus to the anterior touch neurons is needed to evoke a reversal when the posterior touch neurons are also stimulated, compared to when no posterior stimulus is present [27].

**Fig 3:**
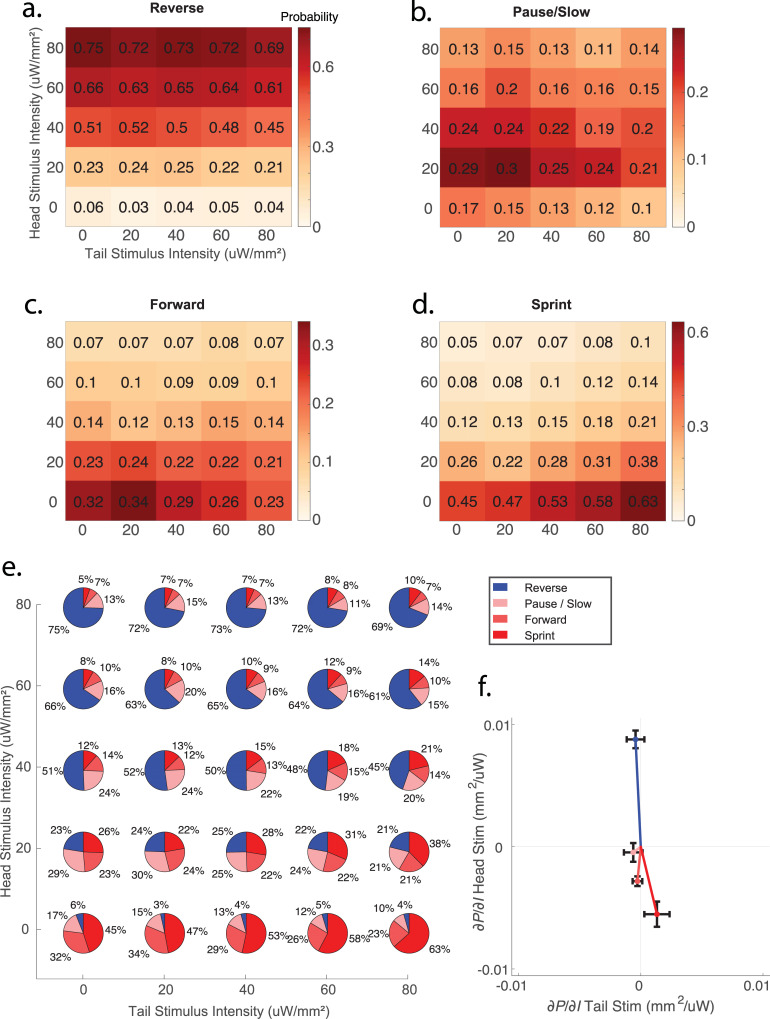
Behavioral response to competing stimulation of anterior and posterior mechanosensory neurons. Various combinations of light intensity was delivered to the head and tail of worms expressing Chrimson in gentle touch mechanosensory neurons (strain AML470, *n* = 43,418 stimulus events total, supserset of data shown in Fig 2). **(a)** Probability of transitioning into reverse, **(b)** pause/slow, **(c)** forward, and **(d)** sprint behaviors are shown individually **(e)** and all together as pie charts. **(f)** The gradient of the plane of best fit is shown as a vector for each behavior. Fitting was performed using methods of least squares. Error-bars are 95% confidence intervals. Numerical values are listed in S2 Data.

To systematically characterize how anterior and posterior mechanosensory signals are integrated, we inspected the animal’s probability of transitioning into reverse, forward, pause/slow, or sprint behavior states in response to 25 different combinations of head and tail light intensity stimuli (Fig 3A–3D). These data are a superset of those shown in Fig 2. Here, 43,418 stimulus events are shown, corresponding to all 25 different conditions. Behavior is defined such that the animal always occupies 1 of 4 states: reverse, pause/slow, forward, or sprint, so that for a given stimulus condition, the four probabilities necessarily sum to one (Fig 3E).

To explore the dependence of the probability of evoking a given behavior on the anterior and posterior stimulus intensity, we fit planes to the probability surfaces and computed the gradient (Fig 3F). Fitting was performed using the method of least squares. The head and tail components of the gradient of the plane serve as a simplified linear approximation of the probability landscape and can provide a succinct estimate of how the probability depends on either head or tail stimulus illumination intensity. For example, the probability of reversal depends strongly on the head stimulus intensity, as indicated by the large positive head component of the gradient. The probability of reversal also depends slightly on the tail stimulus intensity, consistent with [27], but we note this dependence was small and that the 95% confidence intervals of this dependence spanned the zero value. Of the 4 behaviors tested, only sprint behavior depended on both head and tail stimulation intensity such that the 95% confidence intervals of both components of their gradient excluded the value zero. Sprints occurred with highest probability at the highest tail illumination intensity when head illumination was zero. As head illumination increased, the probability of a sprint rapidly decreased. This is captured quantitatively by the gradient plotted in Fig 3F, which shows that the ratio of the dependence of the sprint probability on tail versus head stimulation is roughly 1:−4. One interpretation is that head induced reversals are less likely to be counteracted by a tail stimulation, than tail induced sprints are to be counteracted by head stimulation.

Taken together, we conclude that anterior and posterior mechanosensory signals are combined to determine the relative likelihood of different behavioral responses. Sprinting behavior is sensitive to both stimuli in a −4 to 1 ratio, while other behaviors such as reversals depend overwhelmingly more on one stimulus (head stimulation) than the other (tail stimulation). This places constraints on any quantitative models of sensory integration performed by downstream circuitry.

### Behavior-triggered stimulation increases throughput when investigating rare behaviors

*C*. *elegans*’ response to mechanosensory stimulation depends on its behavioral context. The probability that a tap or optogenetic mechanosensory stimulation evokes a reversal is higher when the stimulus occurs as the animal moves forward compared to if the stimulus occurs when the animal is in the midst of a turn, suggesting that the nervous system gates mechanosensory evoked responses during turns [48]. This was first observed in open-loop experiments in which the animal was stimulated irrespective of its behavior. Those experiments relied on identifying, after the fact, stimulus events that arrived by chance during turning. Because turning events are brief and occur infrequently, it can be challenging to observe sufficient numbers of stimuli events delivered during turn onset using such an open-loop approach. For example, in that work, only 15 optogenetic stimulus events for a given stimulus intensity condition landed during turns. The animal’s spontaneous rate of turn initiation varies with the presence of food and other environmental conditions, but it has been reported to be approximately 0.03 Hz [64].

To obtain higher efficiency and throughput at probing the animal’s response to stimulation during turning, we delivered closed-loop stimulation automatically triggered on the onset of the animal’s turning behavior. Full-body red light illumination (1.5-mm diameter) was automatically delivered to animals expressing Chrimson in the gentle touch mechanosensory neurons (strain AML67, same as in [48]) when the real-time tracking software detected that the animal entered a turn. Turn onset was detected by monitoring the ellipse ratio of a binary image of the animal, as described in Materials and methods. A refractory period of 30 seconds was imposed to prevent the same turning event from triggering multiple stimuli and served to set a minimum interstimulus interval. A total of 47 plates of animals were recorded for 30 minutes each over 3 days, and on average, the system simultaneously tracked or stimulated 44.5 ± 20 worms on a plate at any given time (S1 Table). Three different stimulus intensities (0.5, 40, and 80 uW/mm^2^) and three different stimulus durations (1, 3, and 5 seconds) were explored, totaling 22,608 turn-triggered stimuli events delivered to valid worms, of which on postprocessing analysis 9,776 or 43.2% passed our more stringent inclusion criteria for turn onset, worm validity, and track stability (Table 1). To compare the closed- and open-loop approaches, 29 additional plates were stimulated in open loop over the same 3-day period.

**Table 1:** Comparison of open- and closed-loop approaches for studying the animal’s response to stimulation during a turn.

Ref.	Experiment type	Plates	Cum. recording duration (worm hour)	ISI (seconds)	All stim events on valid worms	Turn-associated stim events	Yield	Throughput (turn-associated stim events/worm hour)
This work	Closed-loop optogenetic	47	1,060	>30	22,608	9,776	43.2%	9.2
This work	Open-loop optogenetic	29	633	30	41,623	308	0.7%	0.5
[48]	Open-loop optogenetic	12	260	60	2,487	15	0.6%	0.35*

**Fig 4:**
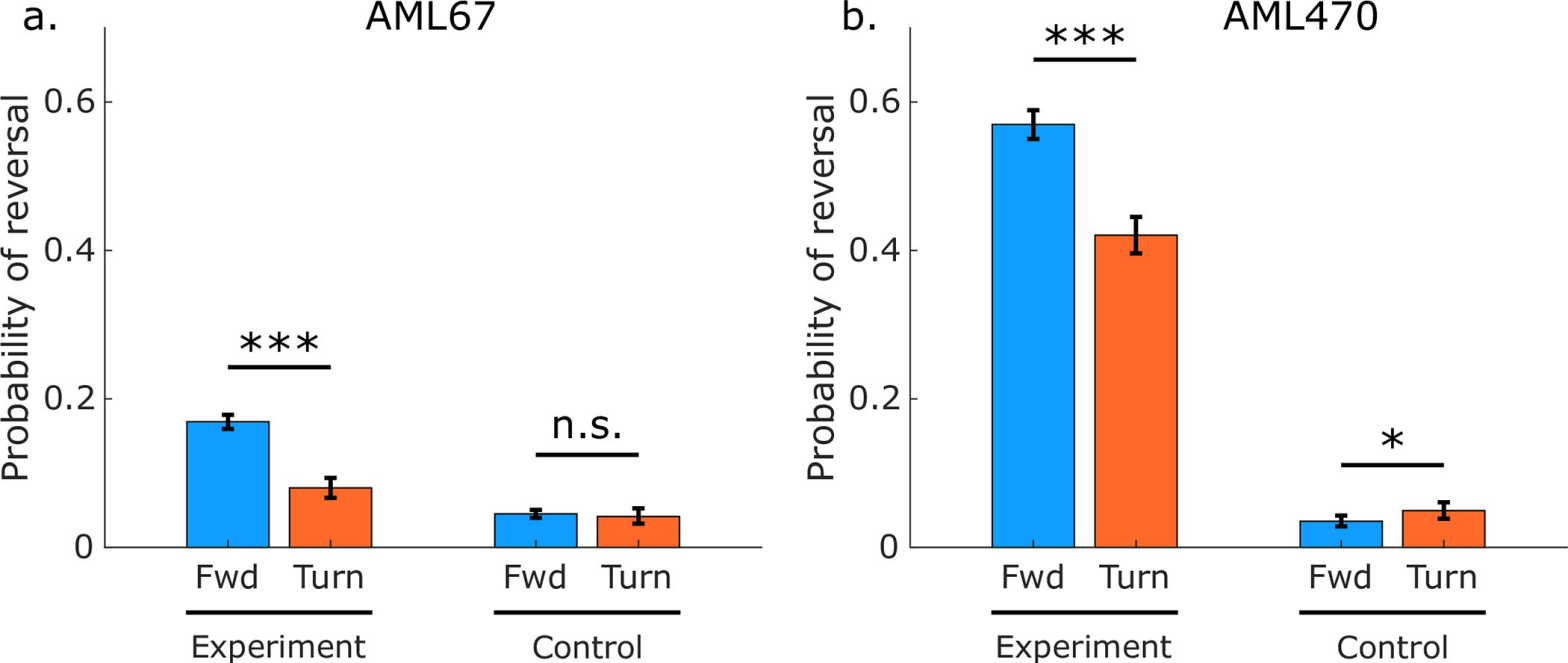
Probability of reversing in response to a mechanosensory stimulus is higher for stimuli that arrive during forward locomotion than for stimuli that arrive during turning. Response to whole body optogenetic illumination of gentle touch mechanosensory neurons is shown for stimuli that arrive during either forward or turning behaviors for 2 strains of nominally identical genotypes, **(a)** AML67 and **(b)** AML470. Stimuli delivered during turns are from closed-loop optogenetic experiments, while stimuli delivered during forward locomotion are from open-loop experiments. Three seconds of 80 uW/mm^2^ illumination was delivered in the experiment condition. Only 0.5 uW/mm^2^ was delivered for control condition. Error bars are 95% confidence interval calculated via 10,000 bootstraps. Z-test was used to calculate significance. *** indicates *p*<0.001. *p*-Value for AML67 control group is 0.549. *p*-Value for AML470 control group is 0.026. The number of stimulus events for each condition (from left-most bar to right-most bar) are 5,968, 1,551, 5,971, and 1,448 for AML67 and 2,501, 1,543, 2,676, and 1,438 for AML470. Machine-readable numerical values are listed in S3 and S4 Data.

Whole-body illumination experiments using AML67 are shown. This is a superset of the data shown in Fig 4 and includes a variety of stimulus intensities and stimulus durations. Compared to an open-loop approach, closed-loop turn-triggered stimulation provides higher throughput and higher yield.

*Note that we report an effective throughput for experiments in [48] to account for discrepancies in how different stimulus intensities are reported in that work (reported cumulative worm hours include six different stimuli intensities, while only one intensity is considered in the reported number of stimulus events).

ISI, interstimulus interval.

We compared the probability of reversing in response to closed-loop stimuli delivered during turn onset against the probability of reversing in response to open-loop stimuli delivered during forward locomotion (Fig 4A). For this analysis, we considered only stimuli of 3-second duration and either 80 uW/mm^2^ or 0.5 uW/mm^2^ (control) illumination intensity. A total of 2,999 stimulus events of this duration and intensities were delivered during turn onset and met our inclusion criteria. Consistent with previous reports, the animal was significantly more likely to initiate a reversal in response to stimuli delivered during forward locomotion than during turning. We repeated this experiment in strain AML470. That strain was also statistically significantly more likely to reverse in response to stimuli delivered during forward locomotion than during turning, in agreement with our prior findings. Interestingly, the effect was less striking in this strain compared to AML67 even though animals were overall more responsive. One possible interpretation is that turning-induced inhibition is in competition with mechanosensory signals. Because AML470 is apparently more sensitive to stimulation than AML67, it may also be more likely to generate stronger mechanosensory signals that overcome turning-induced inhibition, consistent with findings in Fig 4. By using a high-throughput closed-loop approach, we achieved larger sample size (2,999 events for a single strain, intensity, and control; compared to 15 [48]), confirmed previous findings, and refined our understanding of how turning-induced inhibition may compete with mechanosensory signals.

Both throughput and efficiency are relevant for studying stimulus response during turning. Throughput refers to the number of stimuli delivered during turns per time. High throughput is needed to generate a large enough sample size in a reasonable enough amount time to draw statistically significant conclusions. Efficiency, or yield, refers to the fraction of delivered stimuli that were indeed delivered during turns. A high efficiency, or yield, is desired to avoid unwanted stimulation of the animal, which can lead to unnecessary habituation (S5 Fig).

We compared the throughput and efficiency of stimulating during turn onset with closed-loop stimulation to an open-loop approach on the same instrument using our same analysis pipeline and inclusion criteria (Table 1). Again, we considered only stimuli delivered within a small 0.33-second window corresponding to our definition of the onset of turns and applied in postprocessing the same stringent inclusion criteria to both open-loop and closed-loop stimuli. Closed-loop stimulation achieved a throughput of 9.2 turn onset–associated stimulation events per worm hour, an order of magnitude greater than the 0.5 events per worm hour in open loop stimulation. Crucially, closed-loop stimulation was also more efficient, achieving a yield of 43.2%, more than 50-fold higher than the 0.7% open-loop yield. We reach similar conclusions by comparing to previous open-loop optogenetic experiments from [48] that had a longer interstimulus interval. Compared to that work, this system achieved a 25-fold increase in throughput and a more than 50-fold increase in yield. Taken together, by delivering stimuli triggered on turns in a closed-loop fashion, we achieved higher throughput and efficiency than open-loop approaches.

### Further characterization of the instrument

We sought to further characterize the system to better understand its capabilities and limitations. We quantified metrics of latency, dropped frames, and spatial drift by analyzing the recordings related to anterior and posterior stimulation (S1 Fig) and the recordings related to turning (S2 Fig).

The closed-loop latency between an animal’s movement and a corresponding change in illumination output based on that movement is a key determinant of system accuracy. If that latency is too high compared to the animal’s motion, the stimulus will arrive off-target. We estimate the total closed-loop latency by summing two measured latencies: the first is the latency measured via the projected timestamps associated with acquiring an image, drawing a stimulus and projecting that stimulus (acquire–draw–project, S1A Fig). The second is the recorded latency of the time to segment and track worms in a frame (tracking update time or frame rate, S1B Fig). For experiments in Fig 3, the total closed-loop latency was 200 ms or less for more than 95% of the frames in the recording. For a typical worm moving at 200 um/s, this corresponds to a roughly 40 um bound on spatial resolution—sufficient for the 500-um diameter head–tail illuminations used here, but notably longer than the 29-ms latency reported for a single-worm system [29].

Latency increases with the number of worms tracked simultaneously. Turning experiments in Fig 4 used more animals (mean 39 tracked worms in the field of view per frame compared to 12) and consequently had longer estimated roundtrip latencies (S2A and S2B Fig), although still typically less than 300 ms. While appropriate for the stimuli used here, the measures of latency demonstrate that the current system is unlikely to be well suited to target the dorsal versus ventral side of the animal, for example.

## Discussion and conclusions

The approach here dramatically improves throughput in two ways compared to previous methods. First, this work extends targeted illumination to many worms in parallel, providing an order of magnitude higher throughput per plate compared to previous single-worm methods capable of delivering optogenetic illumination targeted to different regions of the body [8,26,27]. Second, the method enables automatic stimulus delivery triggered on a behavior. For studying stimulus response during rare behaviors, like turns, this closed-loop approach provides higher throughput and efficiency compared to previous open-loop methods [48].

To achieve simultaneous independent targeting of many animals and tracking of behavior, we developed and optimized new algorithms, such as real-time centerline tracking algorithms, improved existing ones using parallelization, and also leveraged advances in computing power. For example, we took advantage of the availability of powerful fast 32-core CPUs, multiterabyte solid-state drives, and low latency USB3 cameras. We suspect that both algorithmic advances and increased computer power were necessary.

To attain such high throughput, the system also sacrifices some spatial resolution compared to previous single-worm approaches [8,26,27]. For example, the roundtrip latency and observed drift in calibration places a roughly 100-um floor on our spatial resolution, which makes the system ill-suited for resolving individual neurons located close to one another. At its current field of view, the system is also unlikely to work well with larvae, which are significantly smaller than adults. Nonetheless, the system’s resolution is more than sufficient to selectively illuminate the head or tail of adult *C*. *elegans*, which allows for new types of investigations. For example, we used the instrument to systematically probe anterior–posterior integration of mechanosensory signals for a range of competing stimuli intensities, delivering over 43,418 stimulus events to valid worms. The sample size needed for such an experiment would be impractical with single-worm targeted illumination methods. And current genetic labeling approaches preclude this experiment from being conducted with nontargeted whole field-of-view illumination setups, such as in [48].

Our measurements suggest that the worms’ behavioral response to competing mechanosensory stimuli depends on integrating anterior and posterior mechanosensory signals. A plane roughly captured how the likelihood of a behavior response depended on the intensity of the anterior and posterior stimuli. For example, the probability of a sprint was influenced by signals in both anterior and posterior mechanosensory neurons roughly by a −4 to 1 ratio, while the probability of reversing is determined almost entirely by the anterior mechanosensory neurons. One interpretation of these measurements is that head stimuli that would induce reversals are less likely to be counteracted by a tail stimulation, than tail induced sprints are to be counteracted by head stimulation. The *C*. *elegans* response to anterior mechanosensory stimuli is an important part of the escape response [65] and helps the animal avoid predation by nematophagous fungi [66]. It is possible that the relative difficulty in disrupting head induced reversals compared to sprints reflects the relative importance of the role of the reversal in this escape behavior.

Optogenetics allowed us to deliver competing stimuli of varied intensities to the head or tail of a moving worm. Because optogenetic stimulation bypasses the animal’s mechanotransduction channels and further depends on the relative expression pattern of Chrimson, one may ask whether the animal perceives optogenetic stimulation similarly to mechanical stimulation. We previously observed that optogenetic stimulation of all 6 gentle touch mechanosensory neurons elicited similar responses to that of a wild-type (WT) worm undergoing plate tap [48], suggesting in that case that optogenetic stimulation mimics mechanical stimulation. Since no current method is capable of delivering precisely varying competing mechanical stimuli to moving animals, we were not able to directly compare optogenetic and mechanosensory stimuli for the competing anterior–posterior case.

Here, we used red illumination to excite Chrimson, but we note that the system can be trivially extended to independently deliver simultaneous red and blue light illumination [27], for example, to independently activate two different opsins such as the excitatory red opsin Chrimson and the inhibitory blue opsin gtACR2 [67,68]. Like other targeted illumination systems before it [26,27], this system is not capable of targeting regions within the body when the animal touches itself, as often occurs during turning, or when coiling [69,70]. This still permits probing the animal’s response to mechanosensory stimulation during turns because we were interested in whole-animal stimulation for those specific experiments, rather than targeting the head or tail. We note that our postprocessing analysis does resolve the animal’s centerline even during self-touching [48], but that method is not currently suitable for real-time processing.

We investigated the response to stimulus during turning by delivering closed-loop stimuli automatically triggered on the turn. We achieved a more than 25-fold increase in throughput compared to a previous investigation [48] and similar order of magnitude increase compared to an open-loop approach implemented on the same instrument with the same analysis pipeline and inclusion criteria. The closed-loop functionality can be easily triggered on sprints or pauses or, in principal, even on extended motifs like an escape response. This high-throughput triggering capability may be useful for searching for long-lived behavior states, probing the hierarchical organizations of behavior [71] or exploring other instances of context-dependent sensory processing [48].

## Materials and methods

### Strains

Two strains were used in this work, AML67 and AML470, described in Table 2. A list of experiments and figures cross-referenced by strain is shown in S1 Table. Both strains expressed the light-gated ion channel Chrimson and a fluorescent reporter mCherry under the control of a *mec-4* promoter and differed by the concentration of the Chrimson-containing plasmid used for injection, as well as by the background and degree of outcrossing. AML67 was injected with 40 ng/ul of the Chrimson-containing plasmid, while AML470 was injected with 10 ng/ul. Specifically, to generate AML470, a plasmid mix containing 10 ng/ul of pAL::pmec-4::Chrimson::SL2::mCherry::unc-54 (RRID:Addgene_107745) and 100 ng/ul of pCFJ68 unc-122::GFP (RRID:Addgene_19325) were injected into CZ20310 [72] and then integrated via UV irradiation. Experiments were conducted with AML470 strains prior to outcrossing. We note that AML470 animals appear slightly smaller than similarly aged animals of strain AML67 or WT.

**Table 2:** Strains used.

Strain	RRID	Genotype	Notes	Ref.
AML67	RRID:WB-STRAIN:WBStrain00000193	wtfIs46[pmec-4::Chrimson::SL2::mCherry::unc-54 40ng/ul]	40-ng Chrimson injection	[48]
AML470		juSi164[mex-5p::HIS-72::miniSOG+Cbr-unc-119(+)] unc-119(ed3) III; wtfIs458 [mec-4::Chrimson::SL2::mCherry::unc-54 10 ng/ul + unc-122::GFP 100 ng/ul]	10-ng Chrimson injection	This work

### Instrument

#### Hardware

A CMOS camera (acA4112-30um, Basler, Ahrensburg, Germany) captured via global shutter images of worms crawling on a 9-cm diameter agar plate at 30 frames per second, illuminated by a ring of 850-nm infrared LEDs, all housed in a custom cabinet made of 1 inch aluminum extrusions. To illuminate the worm, a custom projector was built by combining a commercial DMD-based light engine (Anhua M5NP, containing a Texas Instruments DLP4500) with a Texas Instrument evaluation control board (DLPLCR4500EVM). The light engine contained red, green, and blue LEDs with peaks at 630 nm, 540 nm, and 460 nm, respectively. The projector cycles sequentially through patterns illuminated by red, green, and then blue illumination once per cycle at up to 60 Hz and further modulates the perceived illumination intensity for each pixel within each color by fluttering individual mirrors with varying duty cycles at 2.8 kHz. The system produced a small image (9-cm wide) from a short distance away (15 cm) such that a single element of the DMD projects light onto a roughly 85 um^2^ region of agar.

A light engine driven by a separate evaluation board was chosen instead of an all-in-one off-the-shelf projector because the application programming interface (API) provided by the evaluation board allowed for more precise timing and control of all aspects of the illumination, including the relative exposure duration and bit depth of the red, green and blue channels. For example, in this work, only the red and green channels are used. So for these experiments, the projector was programmed to update at 30 Hz and display a green pattern for 235 us (1 bit depth), followed by a red pattern for 33,098 us (8 bit depth) during each 30-Hz cycle. This choice of timing and bit depth maximizes the range of average intensities available in the red channel for optogenetic stimulation, while restricting the green channel to binary images sufficient for calibration. The choice of 30 Hz is optimized for the 30-Hz camera framerate. To avoid aliasing, camera acquisition was synchronized to the projector in hardware by wiring the camera trigger input to the green LED on the light engine. If both red and blue channels are to be used for optogenetic stimulation, a different set of timing parameters can be used.

It is desired that the animal perceives the illumination as continuous and not flickering. The inactivation time constant for Chrimson is 21.4±1.1 ms [73]. As configured, the 80 uW/mm^2^ illumination intensity generates a gap in red light illumination of only 235 us from cycle to cycle, well below Chrimson’s inactivation time constant. Therefore, the animal will perceive the illumination as continuous. At 20 uW/mm^2^, the gap in illumination due to the temporal modulation of the micromirrors is nearly 25 ms, similar to the inactivation timescale. Intensities lower than this may be perceived as flickering. The only lower intensities used in this work were 0.5 uW/mm^2^, for certain control experiments. We were reassured to observe no obvious behavioral response of any kind to 0.5 uW/mm^2^ illumination, suggesting that in this case the animal perceived no stimulus at all.

A set of band-pass and long-pass filters was used in front of the camera to block red and blue light from the projector while passing green light for calibration and IR light for behavior imaging. These were, in series, a 538/40 nm band-pass filter (Semrock (West Henrietta, New York), FF01-538/40-35-D), a 550-nm long-pass filter (Schott (Mainz, Germany), OG-550), and 2 color photography filters (Roscolux (Stamford, Connecticut), #318). A 16-mm c-mount lens (Fujinon (Tokyo, Japan), CF16ZA-1S) and spacer (CMSP200, Thorlabs, Newton, New Jersey) was used to form an image. Barrel distortion is corrected in software.

A PC with a 3.7 GHz CPU (AMD 3,970×) containing 32 cores and a GPU (Quadro P620, Nvidia, Santa Clara, California) controlled the instrument and performed all real-time processing. A 6 TB PCIe solid-state drive provided fast writeable storage on which to store high-resolution video streams in real time (Table 3). Images from the camera arrived via USB-C. Drawings were sent to the projector’s evaluation board via HDMI.

**Table 3:** Input and output video streams used or generated by the instrument.

Video stream	Resolution	Format	Bandwidth (MB/s)
Camera video in (real time)	2048 × 1504 at 30 Hz	8-bit monochrome via USB-C	85.3
Camera video saved (real time)	2048 × 1504 at 30 Hz	8-bit monochrome TIFF	85.3
Camera video compressed (postprocessing)	2048 × 1504 at 30 Hz	8-bit monochrome HEVC	0.102
Projector video out (real time)	912 × 1140 at 60 Hz	8-bit RGB* via HDMI	178
Projector video saved (real time)	912 × 570 at 30 Hz	8-bit RGB TIFF	44.5
Projector video aligned to camera frame of reference (postprocessing)	2048 × 1504 at 30 Hz	8-bit RGB PNG	0.353

*The green channel is actually displayed as binary since it is only used for calibration. Single color experiments in red or blue can achieve 8-bit color resolution but runs at 30 Hz. For experiments with both red and blue, the projector can only simultaneously decode 7 bits of color resolution for each channel, but it runs at 60 Hz.

Bandwidth is reported in megabytes per second of the recording.

A complete parts list is provided in S2 Table, and additional details are described in [74].

#### Real-time software

**Fig 5:**
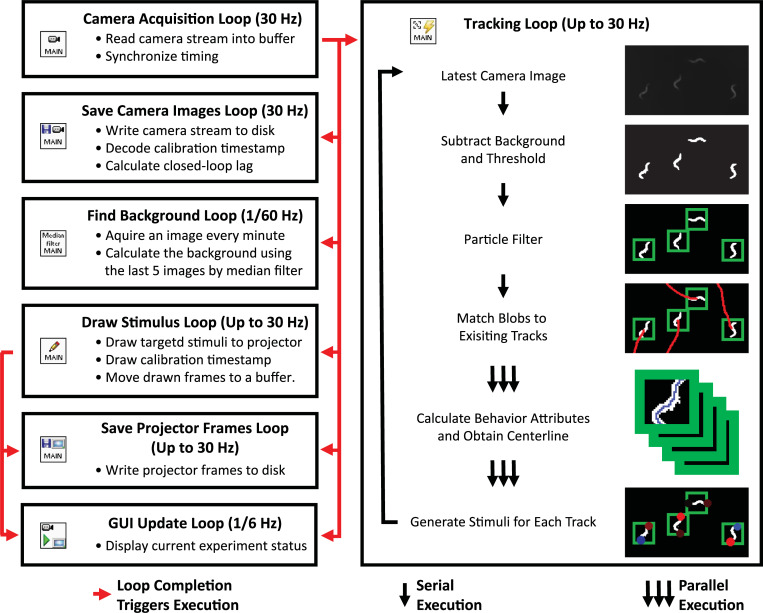
Selected software modules involved in closed-loop light stimulation. Selected software modules are shown that run synchronously or asynchronously. Note that the image processing depiction is illustrative and for a small cropped portion of the actual field of view.

**Fig 6:**
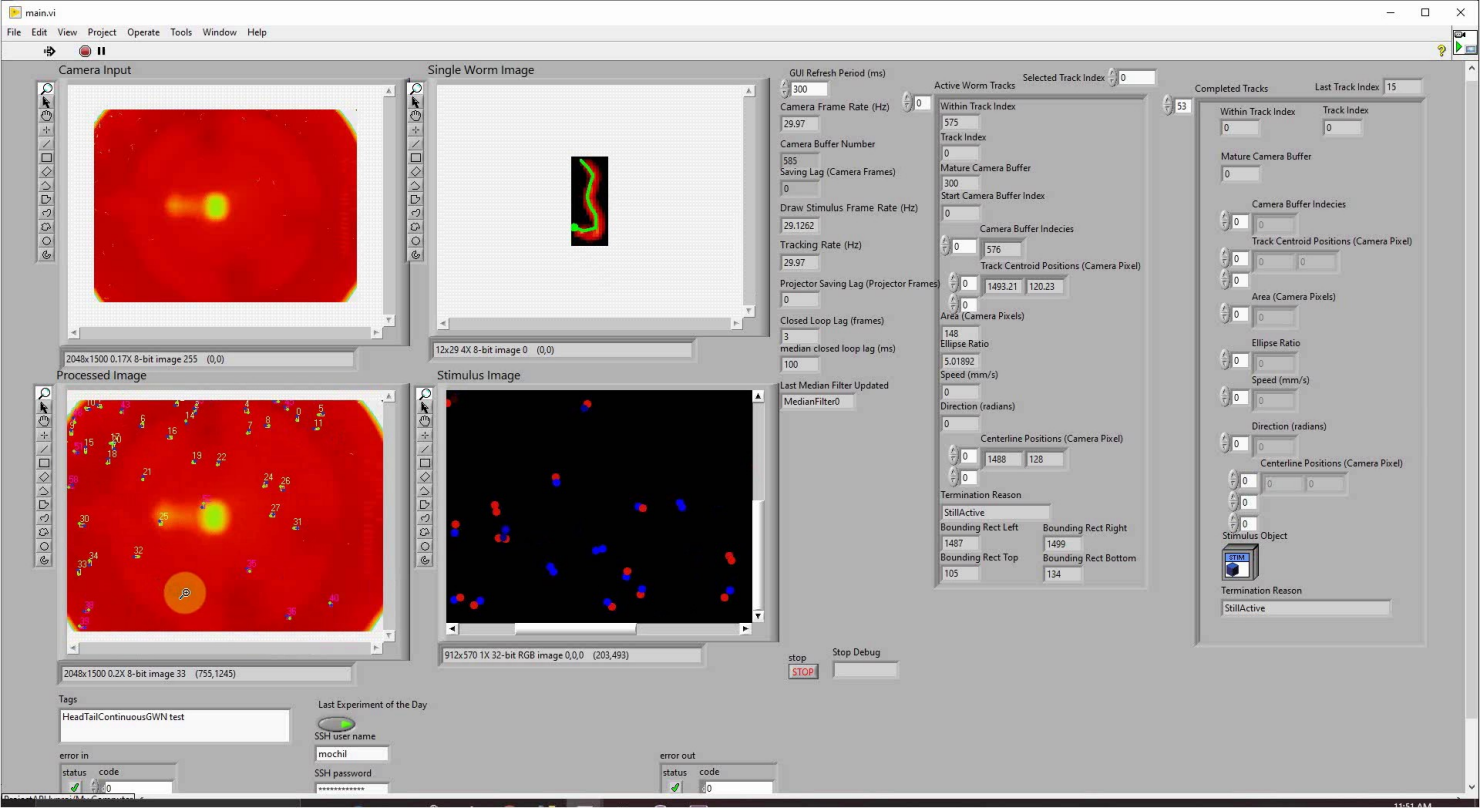
GUI shown here during an experiment. GUI, graphical user interface.

Custom LabVIEW software was written to perform all real-time image processing and to control all hardware. Software is available at https://github.com/leiferlab/liu-closed-loop-code. The LabVIEW software is described in detail in [74]. The software is composed of many modules, summarized in Fig 5. These modules run separately and often asynchronously to acquire images from the camera, track worms, draw new stimuli, communicate with the projector, and update a graphical user interface (GUI) shown in Fig 6. The software was designed with parallel processing in mind and contains many parallel loops that run independently to take advantage of the multiple cores.

Camera images and drawn projector images are both saved to disk in real time as TIFF images (Table 3). In postprocessing, they are compressed and converted to H.265 HEVC videos using the GPU.

**Fig 7:**
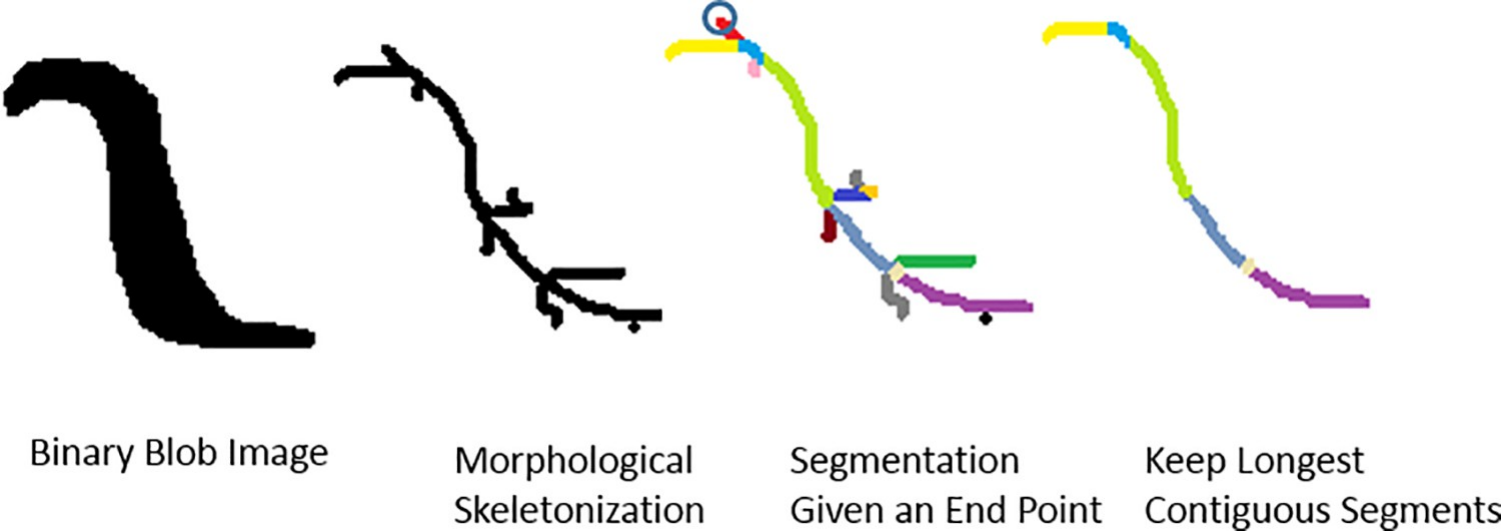
Illustration of the fast centerline finding algorithm. The algorithm proceeds in order from left to right. A binary image of the worm is taken as input. A skeleton is then generated through morphological thinning. The first recursive algorithm starts from an endpoint of the skeleton and breaks it down into segments at each branch point. The second recursive algorithm uses these segments to find the longest contiguous segment from one end point to another end point.

A critical task performed by the software is to track each animals’ centerline in real time so that targeting can be delivered to different regions of the animal. Many centerline algorithms have been developed [47], including some that can operate on a single animal in real time, for example, [26]. The existing algorithms we tested were too slow to run simultaneously on the many animals needed here. Instead, we developed a 2-pass recursive algorithm that is fast and computationally efficient (Fig 7). An image of the worm is binarized and then skeletonized through morphological thinning. Then, in the first pass, the skeleton is traversed recursively to segment all of the distinct branch points in the skeleton. Then in a second pass, the path of the centerline is found by recursively traversing all sets of contiguous segments to identify the longest contiguous path. The longest contiguous path is resampled to 20 points and reported as the centerline. Further details are described in [74].

We note that the extracted centerlines can be slightly shorter than the full length of the animal (visible, for example, in Fig 1C) and that this may contribute to slightly underestimating the true distribution of worm lengths, as in S1 and S2 Figs.

From the animal’s centerline, other key attributes are calculated in real time, including the animal’s velocity and eccentricity, which are used to determine whether the animal is turning. The software also stitches together images of worms into tracks, using methods adopted from the Parallel Worm Tracker [43] and described in [48,74]. To identify the head or tail in real time, the software assumes that the worm cannot go backward for more than 10 seconds [19], and so the direction of motion for greater than 10 seconds indicates the orientation of the head. The software makes a provisional assessment of head–tail orientation in real time based on the animal’s trajectory that it has observed so far. In postprocessing, a more definitive assignment is made based on observing the entire duration of the trajectory.

User-specified illumination patterns are delivered to the animal. The user can specify patterns with respect to the animal’s centerline, such as the anterior or posterior illumination patterns used here. By design, the stimulus spots do not collide with nearby worms because real-time software detects when another worm is encroaching on a stimulus spot and automatically crops the stimulus spot to avoid the encroaching worm.

Although not used in this work, the software also contains built-in routines to generate temporally varying patterns, including white noise flicker stimuli, and routines to generate spatial patterns including gradients that cover the entire field of view.

#### Registration and calibration

To generate a map between locations in a camera image and locations on the agar, images are acquired of a calibration grid of dots. A transformation is then generated that accounts for barrel distortion and other optical aberrations.

To generate a map between projected pixels and the image viewed by the camera, the projector draws a spatial calibration pattern in the green channel before each recording. This projector-generated pattern is segmented in software and automatically creates a mapping between projector and camera image. The calibration is also performed at the end of each recording to quantify any drift that occurred between projector and camera during the course of the recording.

To provide temporal calibration and to measure various contributions to latency, a visual frame stamp is projected in the green channel for every frame. The time stamp appears as a sequence of dots arranged in a circle, each dot representing one binary digit. Any worms inside the circle are all excluded from analysis. For calibration, illumination intensity measurements were taken using a powermeter.

#### Behavior analysis

The system extracts centerlines and velocities from the animals, which can then be used to classify animal behavior using a variety of techniques, including based on the animal’s velocity (Figs 2 or 3) or based on the animal’s pose dynamics (Fig 4).

After images have been compressed in HEVC format, data are sent to Princeton University’s high performance computing cluster, Della, for postprocessing and data analysis. Custom MATLAB scripts based on [48] inspect and classify animal behavior. For example, the real-time centerline algorithm fails when the animal touches itself. Therefore, when analyzing behavior after the fact, a slower centerline algorithm [75], as implemented in [48], is run to track the centerline even through self-touches.

The animal’s velocity is smoothed with 1-second boxcar window and used to define behavior states for the experiments in Figs 2 and 3 according to equal area cutoffs shown in S3 Fig.

The turning investigation in Fig 4 uses the behavior mapping algorithms from [48] to classify behavior based on a frequency domain analysis of the animal’s pose dynamics. This is based on an approach first described in [76]. The mapping approach classifies the behavior state that the animal occupies at each instance in time. In many instances, the classification system decides that the worm is not in a stereotyped behavior, and, therefore, it declines to classify the behavior. We inspect even the unclassfied behaviors and classify the animal as turning if (a) the behavior mapping algorithm from [48] defines it as a turn; or (b) if the behavior mapping algorithm classifies it as a nonstereotyped behavior that has an eccentricity ratio less than 3.6. In this way, we rescue a number of instances of turning that had been overlooked. For turning experiments in Fig 4, additional criteria are also used to determine whether a stimulus landed during the turning onset, and to classify the animal’s behavioral response, as described below.

### Nematode handling

Worm preparation was similar to that in [48]. To obtain day 2 adults, animals were bleached 4 days prior to experiment. To obtain day 1 adults, animals were bleached 3 days prior to experiments. For optogenetic experiments, bleached worms were placed on plates seeded with 1 ml of 0.5 mM ATR mixed with OP50 *Escherichia coli*. Off-retinal control plates lacked ATR. Animals were grown in the dark at 20°C.

To harvest worms for high-throughput experiments, roughly 100 to 200 worms were cut from agar, washed in M9, and then spun down in a 1.5-ml micro centrifuge tube. For imaging, 4 small aliquots of worms in M9 were deposited as droplets on the cardinal directions at the edge of the plate. Each droplet typically contained at least 10 worms for a total of approximately 30 to 50 worms. The droplet was then dried with a tissue.

### Anterior versus posterior stimulation experiments

Day 2 adults were used. Every 30 seconds, each tracked animal was given a 500-um diameter red light stimulation to either the head, tail, or both simultaneously. The illumination spot was centered on the tip of the head or tail, respectively. The stimulus intensity was randomly drawn independently for the head and the tail from the set of 0, 20, 40, 60, and 80 uW/mm^2^ intensities.

To compare behavior state occupancy before and after stimuli in Fig 2, the fraction of all animals in each state was tabulated 2 seconds before the onset of stimulus and precisely at the end of the stimulus. Moreover, 95% confidence intervals were found by bootstrapping the observed before and after samples 1,000 times. Significance was determined by a *p*-value < 0.05, as calculated by a Wilcoxon rank sum test comparing the behavior frequency before and after the stimulus.

Transition probabilities reported in Fig 3 were reported by tabulating, for each stimulus event, the animal’s behavior precisely at the end of the stimulus.

Only those stimuli that land on a valid worm are considered. To be valid, a worm is required to be no more than 700 um in length, have a reasonable shape, and be tracked 8 seconds before and 8 seconds after stimulus onset.

### Mechanosensory evoked response during turning versus forward locomotion

#### Open-loop whole-body illumination experiments

Day 1 adults were used. Every 30 seconds, each tracked worm received a 3-second duration 1.5-mm diameter red light illumination spot centered on its body with illumination intensity randomly selected to be either 80 uW/mm^2^ or 0.5 uW/mm^2^. For a subset of plates, additional illumination intensities were also used, and/or 1 second and 5-second stimulus duration were also used, but those stimulus events were all excluded from analysis in Fig 4. But stimuli of all intensities and duration were counted for the purposes of throughput calculations in Table 1, so long as they passed our further criteria for turning onset, worm validity and track stability described in the next section.

Open-loop stimulation was used to study response to stimulation of animals during forward locomotion. Therefore, only stimulus events that landed when the animal exhibited forward locomotion were included. Forward locomotion was defined primarily by the behavior mapping algorithm, but we also required agreement with our turning onset detection algorithm, described below.

During postprocessing, the following inclusion criteria were applied to select only those stimuli that landed on “valid” worms. Tracking was required to persist for 17 seconds prior to stimulation and for the duration of the stimulation.

Instances when the worm collided with another worm before, during, or immediately after stimulation or stimulations to worms that were stationary, exceedingly fat, oddly shaped, or were shorter than expected (less than 550 um for these experiments) were excluded.

#### Closed-loop turn-triggered whole-body illumination experiments

Whenever the real-time software detected that a tracked worm exhibited the onset of a turn, it delivered 3 seconds of 1.5-mm diameter red light illumination centered on the worm with an intensity randomly selected to be either 80 uW/mm^2^ or 0.5 uW/mm^2^. A refractory period was imposed to prevent the same animal from being stimulated twice in less than a 30-second interval.

Stimulus delivery was triggered by real-time detection of turning onset by triggering on instances when the ellipsoid ratio of the binarized image of the worm crossed below 3.5. During postprocessing, a more stringent set of criteria was applied. To be considered a turn onset, the stimulus was required to land also when the improved behavior mapping pipeline considered the animal to be in a turn. We also required that the stimuli did indeed fall within a 0.33-second window immediately following the ellipse ratio crossing, to account for possible real-time processing errors. We had also observed some instances when tail bends during reversals were incorrectly categorized as turning onset events. We therefore required that turn onsets occur only when the velocity was above −0.05 mm/s in a 0.15-second time window prior to stimulus onset. Finally, the same exclusion criteria regarding worm validity and tracking stability from the open-loop whole-body illumination experiments were also applied.

#### Calculating probability of reversals

To be classified as exhibiting a reversal in response to a stimulation for experiments shown in Fig 4, the animal’s velocity must decrease below −0.1 mm/s at least once during the 3-second window in which the stimulus is delivered.

## Supporting information

S1 Fig Characterization of key performance metrics for anterior–posterior stimulation experiments. Performance is evaluated for the set of experiments related to anterior posterior mechanosensory stimluation, including those shown in Fig 3. **(a)** Acquire–draw–project latency is the latency associated with acquiring an image, drawing a new stimuli, and projecting that stimuli, as determined by visual time stamps drawn by the projector. **(b)** The probability distribution of update times for tracking worms is plotted on a log axis. Overall closed-loop latency is the sum of the acquire–draw–project latency and the tracking update time. **(c)** Histogram of camera-to-projector spatial drift between start and end of 30 minutes recording for each of 151 plates, as measured by a calibration pattern projected onto the agar. **(d)** Probability distribution of worm lengths for all tracks, as estimated by the extracted centerline of the worm. Only worms with lengths 700 um and above are included for behavioral analysis. **(e)** Probability distribution of the duration of tracks in the dataset. **(f)** The probability distribution of the number of tracked worms at any given time. The mean and standard deviation for this set of recordings is 12±10. Further breakdown of the mean animals per frame is listed in S1 Table. Machine-readable numerical values are listed in S5 Data.
(PDF)

S2 Fig Characterization of key performance metrics for turning context experiments. Performance is evaluated for the set of turning related experiments, including those in Fig 4, details of which are described in S1 Table. Subpanels are organized similarly to S1 Fig. For this set of recordings, 1-day-old animals were used, only worms with lengths 550 um and above were included in behavior analysis, and the mean number of tracked worms in a field of view per frame is 39. Machine-readable numerical values are listed in S6 Data.
(PDF)

S3 Fig Classifying behavior by velocity distribution. For the analyses performed in Figs 2 and 3, we classify animal behavior to be one of four states using the velocity distribution aggregated from both (+)ATR and (-)ATR head/tail stimulation experiments conducted with AML470. The “Reverse” state has a velocity of less than 0. The three states with positive velocity are divided so they are equally likely. The slowest state is “Pause/Slow.” The middle state is “Forward,”and the fastest state is “Sprint.” Machine-readable numerical values are listed in S7 Data. ATR, all-trans-retinal.
(PNG)

S4 Fig Animals that lack the necessary cofactor ATR do not exhibit a behavioral response. AML470 worms grown (-)ATR. The fraction of animals belonging to behavioral states segmented by velocity before and after each stimulus condition: **(a)** no stimulus, **(b)** tail only stimulus, **(c)** head only stimulus, and **(d)** combined head and tail stimulus. The before time point is taken 2 seconds prior to the stimulus onset, and the after time point is taken at the end of the 1-second stimulation. The stimulus is a 1 second long 0.5-mm diameter circular dot centered at the tip of the animal’s head and/or tail with a red intensity of 80 uW/mm^2^ (0 uW/mm^2^ in “No Stimulus” condition). Significance between before and after is determined by *p*-values calculated using Wilcoxon rank sum test. The significance test does not correct for multiple hypothesis testing. Error bars represents 95% confidence intervals estimated using 1,000 bootstraps. Machine-readable numerical values are listed in S8 Data. ATR, all-trans-retinal.
(PDF)

S5 Fig Animals exhibit habituation to repeated optogenetic stimulation during the course of 30 minutes. Open-loop optogenetic experiments are shown for strains AML67 (29 plates) and AML470 (20 plates) for only those stimuli that are 80 uW/mm^2^. Stimulation was delivered for 3 seconds at no more than once per 30 seconds. To aggregate responses across plates, stimulus events were binned into 2 minute intervals and the fraction of stimulus events resulting in reversals was reported. The error bars represent the 95% confidence interval calculated using 10,000 bootstraps. Machine-readable numerical values are listed in S9 Data.
(PDF)

S6 Fig Strain AML67 exhibits unexpected response to optogenetic stimulation of posterior mechanosensory neurons. Anterior targeted stimuli evoke reversals but posterior stimuli unexpectedly reduce sprints for worms expressing Chrimson in the gentle touch mechanosensory neurons in this strain. Strain AML67 grown on retinal (+)ATR. **(a–d)** Select single animal velocity in response to no stimulation, tail only stimulation, head only stimulation, and combined head and tail stimulation respectively. Dotted lines denote the onset and termination of stimulation. The stimulus is a 1 second long 0.5-mm diameter circular dot centered at the tip of the animal’s head and/or tail with a red intensity of 80 uW/mm^2^ (0 uW/mm^2^ in “No Stimulus” condition)^2^. **(e–h)** Randomly selected velocity traces for 20 animals in each stimulus condition are color coded red/blue. The traces are sorted by the mean velocity during the 1-second stimulation window. The arrow indicates the selected velocity trace shown in the corresponding column in **a–d**. **(i–l)** Probability density of all tracked velocities for each stimulus condition. The mean velocity at any given time is overlaid as the black line. The number of stimuli events for this condition are shown below. **(m–p)** The fraction of animals belonging to behavioral states segmented by velocity before and after each stimulus condition. The before time point is taken 2 seconds prior to the stimulus onset, and the after time point is taken at the end of the 1-second stimulation. Significance between before and after is determined by *p*-values calculated using Wilcoxon rank sum test. Error bars represents 95% confidence intervals estimated using 1,000 bootstraps. Machine-readable numerical values are listed in S10 Data.
(PDF)

S1 Table List of all recordings in this work. “Stim events on valid animals” refers to those stimulation events that landed on animals that met our inclusion criteria for a worm, including criteria for worm shape, size, and track duration, and minimum movement. Note that here stimulation events for all stimulation intensities and durations are listed, whereas in many of the figures only a single stimulation intensity duration is reported.
(PDF)

S2 Table Hardware parts list. Hardware parts list for the instrument. The frame is made from 1 inch aluminum extrusions and are not included. The unit count is for each instrument.
(PDF)

S1 Video Video shows excerpt from recording of animals (AML470) crawling on plate and undergoing head and tail stimulation. Yellow numbered “x” indicates a tracked animal, and its track is shown in yellow. Green inset shows a single tracked individual in detail. Green dot indicates the animal’s head. Its centerline is shown in green. Tracked animals’ heads and tails are occasionally stimulated at various intensities, indicated by red. The dynamic circular pattern in the center of the screen is the visual time stamp projected by the projector and is used for temporal calibration.
(MP4)

S2 Video Example responses to control illumination (0 uW/mm^2^). Strain AML470. The instrument performs all real-time processing to illuminate the head and the tail of each animal for 1 second, but no light is actually delivered to the animal. Targeted regions are shown in light blue. A total of 20 stimulation events are shown corresponding to the 20 randomly selected stimulation events shown in Fig 2E. The animal with the yellow square corresponds to the stimulation event shown in Fig 2A and denoted with an arrow in Fig 2E. The animal’s centerline is shown in green. The head of the animal is denoted with a green circle. Note that the field of view is centered on each animal, so the animal may be moving even though it never exits the field of view.
(MP4)

S3 Video Example responses to tail illumination (80 uW/mm^2^). Posterior mechanosensory neurons are activated by illuminating the tail of animals expression Chrimson in the gentle touch mechanosensory neurons (strain AML470). Illumination is indicated by red circles. A total of 20 stimulation events are shown corresponding to the 20 randomly selected stimulation events shown in Fig 2F. The animal with the yellow square corresponds to the stimulation event shown in Fig 2B and denoted with an arrow in Fig 2F. The animal’s centerline is shown in green. The head of the animal is denoted with a green circle. Note that the field of view is centered on each animal, so the animal may be moving even though it never exits the field of view.
(MP4)

S4 Video Example responses to head illumination (80 uW/mm^2^). Similar to S3 Video. A total of 20 stimulation events are shown corresponding to the 20 randomly selected stimulation events shown in Fig 2G. The animal with the yellow square corresponds to the stimulation event shown in Fig 2C.
(MP4)

S5 Video Example responses to simultaneous head and tail illumination (80 uW/mm^2^). Similar to S3 Video. A total of 20 stimulation events are shown corresponding to the 20 randomly selected stimulation events shown in Fig 2H. The animal with the yellow square corresponds to the stimulation event shown in Fig 2D.
(MP4)

S1 Data Machine-readable numerical values associated with Fig 2. (XLSX)

S2 Data Machine-readable numerical values associated with Fig 3. (XLSX)

S3 Data Machine-readable numerical values associated with Fig 4A. (XLSX)

S4 Data Machine-readable numerical values associated with Fig 4B. (XLSX)

S5 Data Machine-readable numerical values associated with S1 Fig. (XLSX)

S6 Data Machine-readable numerical values associated with S2 Fig. (XLSX)

S7 Data Machine-readable numerical values associated with S3 Fig. (XLSX)

S8 Data Machine-readable numerical values associated with S4 Fig. (XLSX)

S9 Data Machine-readable numerical values associated with S5 Fig. (XLSX)

S10 Data Machine-readable numerical values associated with S6 Fig. (XLSX)

## Abbreviations

ATR: all-trans-retinal
GUI: graphical user interface
WT: wild-type

## References

[1] Clark D, Freifeld L, Clandinin T. Mapping and Cracking Sensorimotor Circuits in Genetic Model Organisms. Neuron. 2013;78(4):583–95. doi: 10.1016/j.neuron.2013.05.006 23719159 PMC4023671

[2] Boyden E. A history of optogenetics: the development of tools for controlling brain circuits with light. F1000 Biol Rep. 2011;3. doi: 10.3410/B3-11 21876722 PMC3155186

[3] Fenno L, Yizhar O, Deisseroth K. The development and application of optogenetics. Annu Rev Neurosci. 2011;34:389–412. doi: 10.1146/annurev-neuro-061010-113817 21692661 PMC6699620

[4] Datta SR, Anderson DJ, Branson K, Perona P, Leifer A. Computational Neuroethology: A Call to Action. Neuron. 2019;104(1):11–24. doi: 10.1016/j.neuron.2019.09.038 31600508 PMC6981239

[5] Pereira TD, Shaevitz JW, Murthy M. Quantifying behavior to understand the brain. Nat Neurosci. 2020;23(12):1537–49. doi: 10.1038/s41593-020-00734-z 33169033 PMC7780298

[6] Calhoun AJ, Murthy M. Quantifying behavior to solve sensorimotor transformations: advances from worms and flies. Curr Opin Neurobiol. 2017;46:90–8. doi: 10.1016/j.conb.2017.08.006 28850885 PMC5765764

[7] Nagel G, Brauner M, Liewald JF, Adeishvili N, Bamberg E, Gottschalk A. Light Activation of Channelrhodopsin-2 in Excitable Cells of Caenorhabditis elegans Triggers Rapid Behavioral Responses. Curr Biol. 2005;15(24):2279–84. doi: 10.1016/j.cub.2005.11.032 16360690

[8] Kocabas A, Shen CH, Guo ZV, Ramanathan S. Controlling interneuron activity in Caenorhabditis elegans to evoke chemotactic behaviour. Nature. 2012;490(7419):273–7. doi: 10.1038/nature11431 23000898 PMC4229948

[9] Hernandez-Nunez L, Belina J, Klein M, Si G, Claus L, Carlson JR, et al. Reverse-correlation analysis of navigation dynamics in Drosophila larva using optogenetics. Elife. 2015;4. doi: 10.7554/eLife.06225 25942453 PMC4466337

[10] Gepner R, Skanata MM, Bernat NM, Kaplow M, Gershow M. Computations underlying Drosophila photo-taxis, odor-taxis, and multi-sensory integration. Elife. 2015;4:e06229. doi: 10.7554/eLife.06229 25945916 PMC4466338

[11] Schulze A, Gomez-Marin A, Rajendran VG, Lott G, Musy M, Ahammad P, et al. Dynamical feature extraction at the sensory periphery guides chemotaxis. Elife. 2015;4:e06694. doi: 10.7554/eLife.06694 26077825 PMC4468351

[12] Calabrese RL. In search of lost scent. Elife. 2015;4:e08715. doi: 10.7554/eLife.08715 26080004 PMC4468918

[13] Gordus A, Pokala N, Levy S, Flavell SW, Bargmann CI. Feedback from network states generates variability in a probabilistic olfactory circuit. Cell. 2015;161(2):215–27. doi: 10.1016/j.cell.2015.02.018 25772698 PMC4821011

[14] Claridge-Chang A, Roorda RD, Vrontou E, Sjulson L, Li H, Hirsh J, et al. Writing memories with light-addressable reinforcement circuitry. Cell. 2009;139(2):405–15. doi: 10.1016/j.cell.2009.08.034 19837039 PMC3920284

[15] Cho CE, Brueggemann C, L’Etoile ND, Bargmann CI. Parallel encoding of sensory history and behavioral preference during Caenorhabditis elegans olfactory learning. Elife. 2016;5. doi: 10.7554/eLife.14000 27383131 PMC4935464

[16] Wen Q, Po MD, Hulme E, Chen S, Liu X, Kwok S, et al. Proprioceptive Coupling within Motor Neurons Drives C. elegans Forward Locomotion. Neuron. 2012;76(4):750–61. doi: 10.1016/j.neuron.2012.08.039 23177960 PMC3508473

[17] Donnelly JL, Clark CM, Leifer AM, Pirri JK, Haburcak M, Francis MM, et al. Monoaminergic Orchestration of Motor Programs in a Complex C. elegans Behavior. PLoS Biol 2013;11(4):e1001529. doi: 10.1371/journal.pbio.1001529 23565061 PMC3614513

[18] Kato S, Kaplan HS, Schrödel T, Skora S, Lindsay TH, Yemini E, et al. Global brain dynamics embed the motor command sequence of Caenorhabditis elegans. Cell. 2015;163(3):656–69. doi: 10.1016/j.cell.2015.09.034 26478179

[19] Wang Y, Zhang X, Xin Q, Hung W, Florman J, Huo J, et al. Flexible motor sequence generation during stereotyped escape responses. Elife. 2020;9:e56942. doi: 10.7554/eLife.56942 32501216 PMC7338056

[20] Cande J, Namiki S, Qiu J, Korff W, Card GM, Shaevitz JW, et al. Optogenetic dissection of descending behavioral control in Drosophila. Elife. 2018;7:e34275. doi: 10.7554/eLife.34275 29943729 PMC6031430

[21] Boulin T. Reporter gene fusions. In: elegans Research Community TC, editor. WormBook; 2006. Available from: http://www.wormbook.org/chapters/www_reportergenefusions/reportergenefusions.html. 10.1895/wormbook.1.106.1 PMC4781452 18050449

[22] Pfeiffer BD, Jenett A, Hammonds AS, Ngo TTB, Misra S, Murphy C, et al. Tools for neuroanatomy and neurogenetics in Drosophila. Proc Natl Acad Sci U S A. 2008;105(28):9715–20. doi: 10.1073/pnas.0803697105 18621688 PMC2447866

[23] Jenett A, Rubin GM, Ngo TTB, Shepherd D, Murphy C, Dionne H, et al. A GAL4-Driver Line Resource for Drosophila Neurobiology. Cell Rep. 2012;2(4):991–1001. doi: 10.1016/j.celrep.2012.09.011 23063364 PMC3515021

[24] Guo ZV, Hart AC, Ramanathan S. Optical interrogation of neural circuits in Caenorhabditis elegans. Nat Methods. 2009;6(12):891–6. doi: 10.1038/nmeth.1397 19898486 PMC3108858

[25] Wyart C, Del Bene F, Warp E, Scott EK, Trauner D, Baier H, et al. Optogenetic dissection of a behavioural module in the vertebrate spinal cord. Nature. 2009;461(7262):407–10. doi: 10.1038/nature08323 19759620 PMC2770190

[26] Leifer AM, Fang-Yen C, Gershow M, Alkema MJ, Samuel ADT. Optogenetic manipulation of neural activity in freely moving Caenorhabditis elegans. Nat Methods. 2011;8(2):147–52. doi: 10.1038/nmeth.1554 21240279 PMC3032981

[27] Stirman JN, Crane MM, Husson SJ, Wabnig S, Schultheis C, Gottschalk A, et al. Real-time multimodal optical control of neurons and muscles in freely behaving Caenorhabditis elegans. Nat Methods. 2011;8(2):153–8. doi: 10.1038/nmeth.1555 21240278 PMC3189501

[28] Bath DE, Stowers JR, Hörmann D, Poehlmann A, Dickson BJ, Straw AD. FlyMAD: rapid thermogenetic control of neuronal activity in freely walking Drosophila. Nat Methods. 2014;11(7):756–62. doi: 10.1038/nmeth.2973 24859752

[29] Shipley FB, Clark CM, Alkema MJ, Leifer AM. Simultaneous optogenetic manipulation and calcium imaging in freely moving C. elegans. Front Neural Circuits. 2014;8. doi: 10.3389/fncir.2014.00028 24715856 PMC3970007

[30] Porto DA, Giblin J, Zhao Y, Lu H. Reverse-Correlation Analysis of Mechanosensation Circuit in C. elegans Reveals Temporal and Spatial Encoding. Scientific Reports 9, 5182 (2019). doi: 10.1038/s41598-019-41349-0 30914655 PMC6435754

[31] Dong X, Kheiri S, Lu Y, Xu Z, Zhen M, Liu X. Toward a living soft microrobot through optogenetic locomotion control of Caenorhabditis elegans. Science Robotics. 2021;6(55). doi: 10.1126/scirobotics.abe3950 34193562

[32] Stephens GJ, Johnson-Kerner B, Bialek W, Ryu WS. Dimensionality and Dynamics in the Behavior of C. elegans. PLoS Comput Biol 2008;4(4):e1000028. doi: 10.1371/journal.pcbi.1000028 18389066 PMC2276863

[33] Faumont S, Rondeau G, Thiele TR, Lawton KJ, McCormick KE, Sottile M, et al. An Image-Free Opto-Mechanical System for Creating Virtual Environments and Imaging Neuronal Activity in Freely Moving Caenorhabditis elegans. PLoS ONE. 2011;6(9):e24666. doi: 10.1371/journal.pone.0024666 21969859 PMC3182168

[34] Musso PY, Junca P, Jelen M, Feldman-Kiss D, Zhang H, Chan RC, et al. Closed-loop optogenetic activation of peripheral or central neurons modulates feeding in freely moving Drosophila. Elife. 2019;8:e45636. doi: 10.7554/eLife.45636 31322499 PMC6668987

[35] Adamantidis AR, Tsai HC, Boutrel B, Zhang F, Stuber GD, Budygin EA, et al. Optogenetic Interrogation of Dopaminergic Modulation of the Multiple Phases of Reward-Seeking Behavior. J Neurosci. 2011;31(30):10829–35. doi: 10.1523/JNEUROSCI.2246-11.2011 21795535 PMC3171183

[36] O’Connor DH, Hires SA, Guo ZV, Li N, Yu J, Sun QQ, et al. Neural coding during active somatosensation revealed using illusory touch. Nat Neurosci. 2013;16(7):958–65. doi: 10.1038/nn.3419 23727820 PMC3695000

[37] Clancy KB, Koralek AC, Costa RM, Feldman DE, Carmena JM. Volitional modulation of optically recorded calcium signals during neuroprosthetic learning. Nat Neurosci. 2014;17(6):807–9. doi: 10.1038/nn.3712 24728268 PMC4361947

[38] Grosenick L, Marshel JH, Deisseroth K. Closed-loop and activity-guided optogenetic control. Neuron. 2015;86(1):106–39. doi: 10.1016/j.neuron.2015.03.034 25856490 PMC4775736

[39] Krakauer JW, Ghazanfar AA, Gomez-Marin A, MacIver MA, Poeppel D. Neuroscience Needs Behavior: Correcting a Reductionist Bias. Neuron. 2017;93(3):480–90. doi: 10.1016/j.neuron.2016.12.041 28182904

[40] Stirman JN, Brauner M, Gottschalk A, Lu H. High-throughput study of synaptic transmission at the neuromuscular junction enabled by optogenetics and microfluidics. J Neurosci Methods. 2010;191(1):90–3. doi: 10.1016/j.jneumeth.2010.05.019 20538016 PMC2908193

[41] Lee JB, Yonar A, Hallacy T, Shen CH, Milloz J, Srinivasan J, et al. A compressed sensing framework for efficient dissection of neural circuits. Nat Methods. 2019;16(1):126. doi: 10.1038/s41592-018-0233-6 30573831 PMC6335042

[42] Wu MC, Chu LA, Hsiao PY, Lin YY, Chi CC, Liu TH, et al. Optogenetic control of selective neural activity in multiple freely moving Drosophila adults. Proc Natl Acad Sci U S A. 2014;111(14):5367–72. doi: 10.1073/pnas.1400997111 24706830 PMC3986155

[43] Ramot D, Johnson BE, Berry TL Jr, Carnell L, Goodman MB. The Parallel Worm Tracker: A Platform for Measuring Average Speed and Drug-Induced Paralysis in Nematodes. PLoS ONE. 2008;3(5):e2208. doi: 10.1371/journal.pone.0002208 18493300 PMC2373883

[44] Branson K, Robie AA, Bender J, Perona P, Dickinson MH. High-throughput ethomics in large groups of Drosophila. Nat Methods. 2009;6(6):451–7. doi: 10.1038/nmeth.1328 19412169 PMC2734963

[45] Swierczek NA, Giles AC, Rankin CH, Kerr RA. High-throughput behavioral analysis in C. elegans. Nat Methods. 2011;8(7):592–8. doi: 10.1038/nmeth.1625 21642964 PMC3128206

[46] Gershow M, Berck M, Mathew D, Luo L, Kane EA, Carlson JR, et al. Controlling airborne cues to study small animal navigation. Nat Methods. 2012;9(3):290–6. doi: 10.1038/nmeth.1853 22245808 PMC3513333

[47] Husson SJ. Keeping track of worm trackers. In: elegans Research Community TC, editor. WormBook. WormBook; 2012.Available from: http://www.wormbook.org. 10.1895/wormbook.1.156.1 PMC4781246 23436808

[48] Liu M, Sharma AK, Shaevitz JW, Leifer AM. Temporal processing and context dependency in Caenorhabditis elegans response to mechanosensation. Elife. 2018;7:e36419. doi: 10.7554/eLife.36419 29943731 PMC6054533

[49] DeAngelis BD, Zavatone-Veth JA, Gonzalez-Suarez AD, Clark DA. Spatiotemporally precise optogenetic activation of sensory neurons in freely walking Drosophila. Elife. 2020;9:e54183. doi: 10.7554/eLife.54183 32319425 PMC7198233

[50] Meloni I, Sachidanandan D, Thum AS, Kittel RJ, Murawski C. Controlling the behaviour of Drosophila melanogaster via smartphone optogenetics. Sci Rep. 2020;10(1):17614. doi: 10.1038/s41598-020-74448-4 33077824 PMC7572528

[51] Guilbeault NC, Guerguiev J, Martin M, Tate I, Thiele TR. BonZeb: open-source, modular software tools for high-resolution zebrafish tracking and analysis. Sci Rep. 2021;11(1):8148. doi: 10.1038/s41598-021-85896-x 33854104 PMC8047029

[52] Chalfie M, Sulston J. Developmental genetics of the mechanosensory neurons of Caenorhabditis elegans. Dev Biol. 1981;82(2):358–70. doi: 10.1016/0012-1606(81)90459-0 7227647

[53] Chalfie M, Sulston JE, White JG, Southgate E, Thomson JN, Brenner S. The neural circuit for touch sensitivity in Caenorhabditis elegans. J Neurosci. 1985;5(4):956–64. doi: 10.1523/JNEUROSCI.05-04-00956.1985 3981252 PMC6565008

[54] Chiba CM, Rankin CH. A developmental analysis of spontaneous and reflexive reversals in the nematodeCaenorhabditis elegans. J Neurobiol. 1990;21(4):543–54. doi: 10.1002/neu.480210403 2376729

[55] Chalfie M, Hart AC, Rankin CH, Goodman MB. Assaying mechanosensation. In: elegans Research Community TC, editor. WormBook; 2014.Available from: 10.1895/wormbook. PMC4448936 25093996

[56] Wicks SR, Rankin CH. Integration of mechanosensory stimuli in Caenorhabditis elegans. J Neurosci. 1995;15(3 Pt 2):2434–44. doi: 10.1523/JNEUROSCI.15-03-02434.1995 7891178 PMC6578104

[57] Petzold BC, Park SJ, Mazzochette EA, Goodman MB, Pruitt BL. MEMS-based force-clamp analysis of the role of body stiffness in C. elegans touch sensation. Integr Biol. 2013;5(6):853–64. doi: 10.1039/c3ib20293c 23598612 PMC3701114

[58] Mazzochette EA, Fang-Yen C, Goodman MB, Pruitt BL. A Real Time Imaging System for Tracking Freely Moving C. elegans in Touch Assays. In: Microtechnologies in Medicine and Biology. Marina del Rey, CA; 2013.Available from: http://microsystems.stanford.edu/microwiki_upload/4/4b/Mazzochette_MMB.pdf.

[59] Mazzochette EA, Nekimken AL, Loizeau F, Whitworth J, Huynh B, Goodman MB, et al. The tactile receptive fields of freely moving Caenorhabditis elegans nematodes. Integr Biol. 2018;10(8):450–63. doi: 10.1039/c8ib00045j 30027970 PMC6168290

[60] McClanahan PD, Xu JH, Fang-Yen C. Comparing Caenorhabditis elegans gentle and harsh touch response behavior using a multiplexed hydraulic microfluidic device. Integr Biol. 2017;9(10):800–9. doi: 10.1039/c7ib00120g 28914311 PMC5645015

[61] Chalfie M. Neurosensory mechanotransduction. Nat Rev Mol Cell Biol. 2009;10(1):44–52. doi: 10.1038/nrm2595 19197331

[62] Gray JM, Hill JJ, Bargmann CI. A circuit for navigation in Caenorhabditis elegans. Proc Natl Acad Sci U S A. 2005;102(9):3184–91. doi: 10.1073/pnas.0409009101 15689400 PMC546636

[63] Alkema MJ, Hunter-Ensor M, Ringstad N, Horvitz HR. Tyramine Functions independently of octopamine in the Caenorhabditis elegans nervous system. Neuron. 2005;46(2):247–60. doi: 10.1016/j.neuron.2005.02.024 15848803

[64] Pierce-Shimomura JT, Morse TM, Lockery SR. The Fundamental Role of Pirouettes in Caenorhabditis elegans Chemotaxis. J Neurosci. 1999;19(21):9557–69. doi: 10.1523/JNEUROSCI.19-21-09557.1999 10531458 PMC6782915

[65] Pirri JK, Alkema MJ. The neuroethology of C. elegans escape. Curr Opin Neurobiol. 2012;22(2):187–93. doi: 10.1016/j.conb.2011.12.007 22226513 PMC3437330

[66] Maguire SM, Clark CM, Nunnari J, Pirri JK, Alkema MJ. The C. elegans touch response facilitates escape from predacious fungi. Curr Biol. 2011;21(15):1326–30. doi: 10.1016/j.cub.2011.06.063 21802299 PMC3266163

[67] Govorunova EG, Sineshchekov OA, Janz R, Liu X, Spudich JL. Natural light-gated anion channels: A family of microbial rhodopsins for advanced optogenetics. Science. 2015;349(6248):647–50. doi: 10.1126/science.aaa7484 26113638 PMC4764398

[68] Vierock J, Rodriguez-Rozada S, Dieter A, Pieper F, Sims R, Tenedini F, et al. BiPOLES is an optogenetic tool developed for bidirectional dual-color control of neurons. Nat Commun. 2021;12(1):4527. doi: 10.1038/s41467-021-24759-5 34312384 PMC8313717

[69] Croll N. Behavoural analysis of nematode movement. Adv Parasitol. 1975;13:71–122. doi: 10.1016/s0065-308x(08)60319-x 1169872

[70] Croll NA. Components and patterns in the behaviour of the nematode Caenorhabditis elegans. J Zool. 1975;176(2):159–76. doi: 10.1111/j.1469-7998.1975.tb03191.x

[71] Kaplan HS, Salazar Thula O, Khoss N, Zimmer M. Nested Neuronal Dynamics Orchestrate a Behavioral Hierarchy across Timescales. Neuron. 2020;105(3):562–576.e9. doi: 10.1016/j.neuron.2019.10.037 31786012 PMC7014571

[72] Noma K, Jin Y. Rapid Integration of Multi-copy Transgenes Using Optogenetic Mutagenesis in Caenorhabditis elegans. G3 (Bethesda). 2018;8(6):2091–7. doi: 10.1534/g3.118.200158 29691291 PMC5982835

[73] Klapoetke NC, Murata Y, Kim SS, Pulver SR, Birdsey-Benson A, Cho YK, et al. Independent optical excitation of distinct neural populations. Nat Methods. 2014;11(3):338–46. doi: 10.1038/nmeth.2836 24509633 PMC3943671

[74] Liu M. C. elegans behaviors and their mechanosensory drivers. Princeton University: Princeton, United States of America; 2020. Available from: http://arks.princeton.edu/ark:/88435/dsp01tt44pq78z.

[75] Deng Y, Coen P, Sun M, Shaevitz JW. Efficient Multiple Object Tracking Using Mutually Repulsive Active Membranes. PLoS ONE. 2013;8(6):e65769. doi: 10.1371/journal.pone.0065769 23799046 PMC3683037

[76] Berman GJ, Choi DM, Bialek W, Shaevitz JW. Mapping the stereotyped behaviour of freely moving fruit flies. J R Soc Interface. 2014;11(99). doi: 10.1098/rsif.2014.0672 25142523 PMC4233753

