# Supplementary figures and images for: A high-throughput method to deliver targeted optogenetic stimulation to moving C. elegans populations
Source: PLoS Biol. 2022 Jan 28;20(1):e3001524. doi: 10.1371/journal.pbio.3001524 (PMC8827482; doi:10.1371/journal.pbio.3001524)

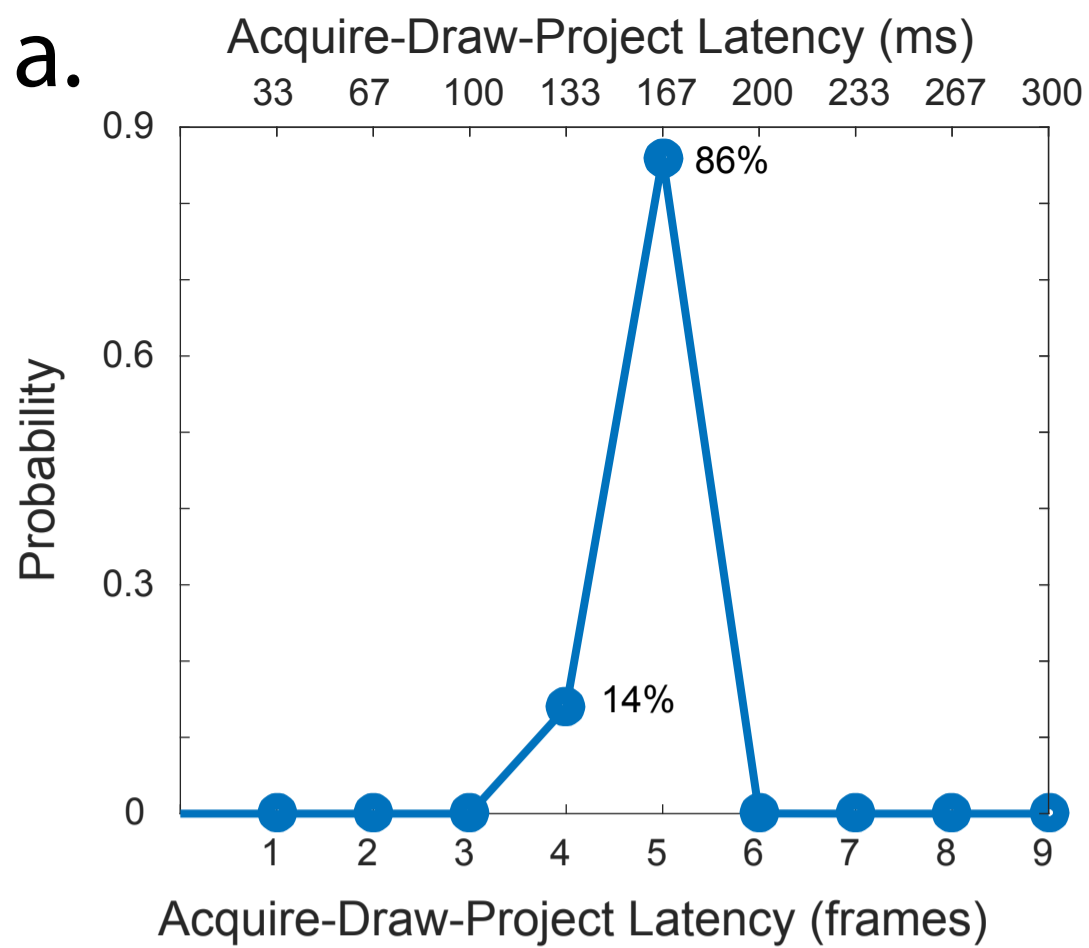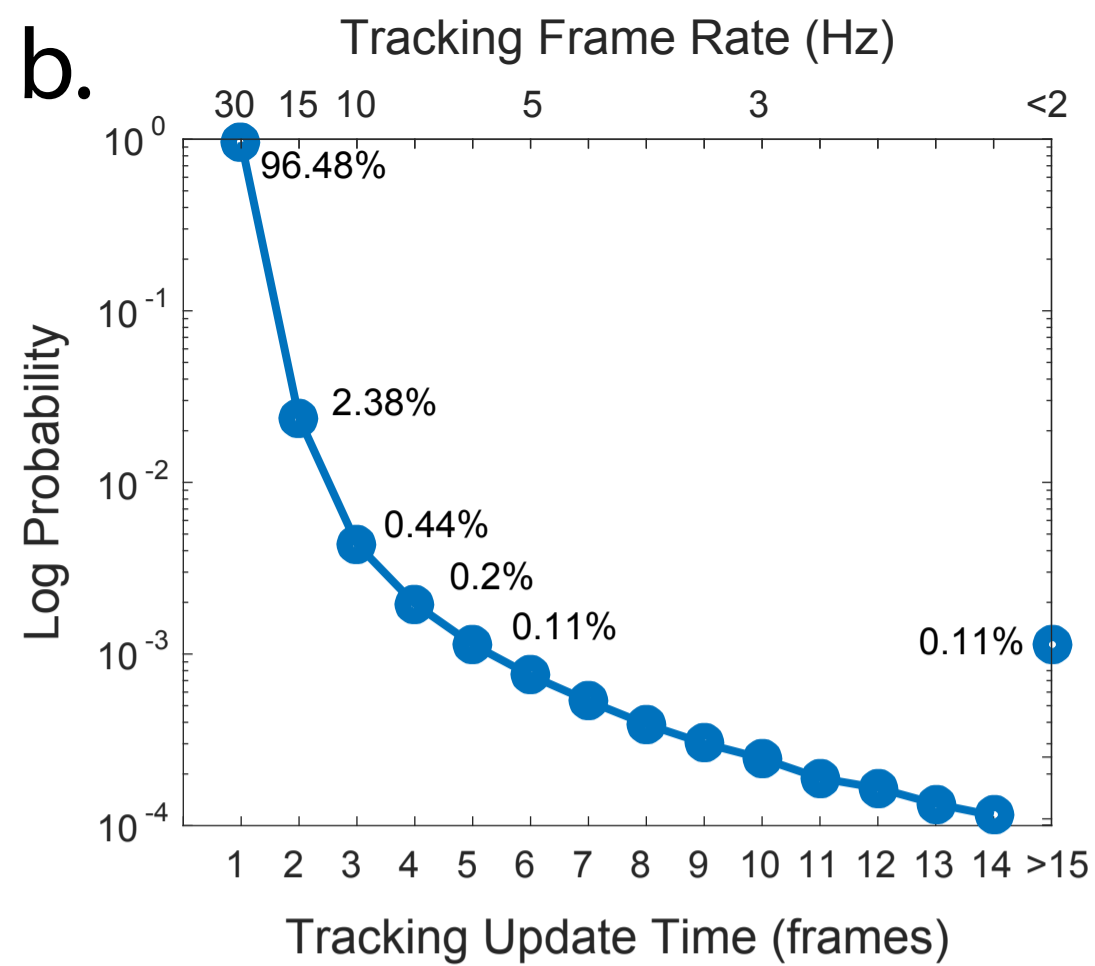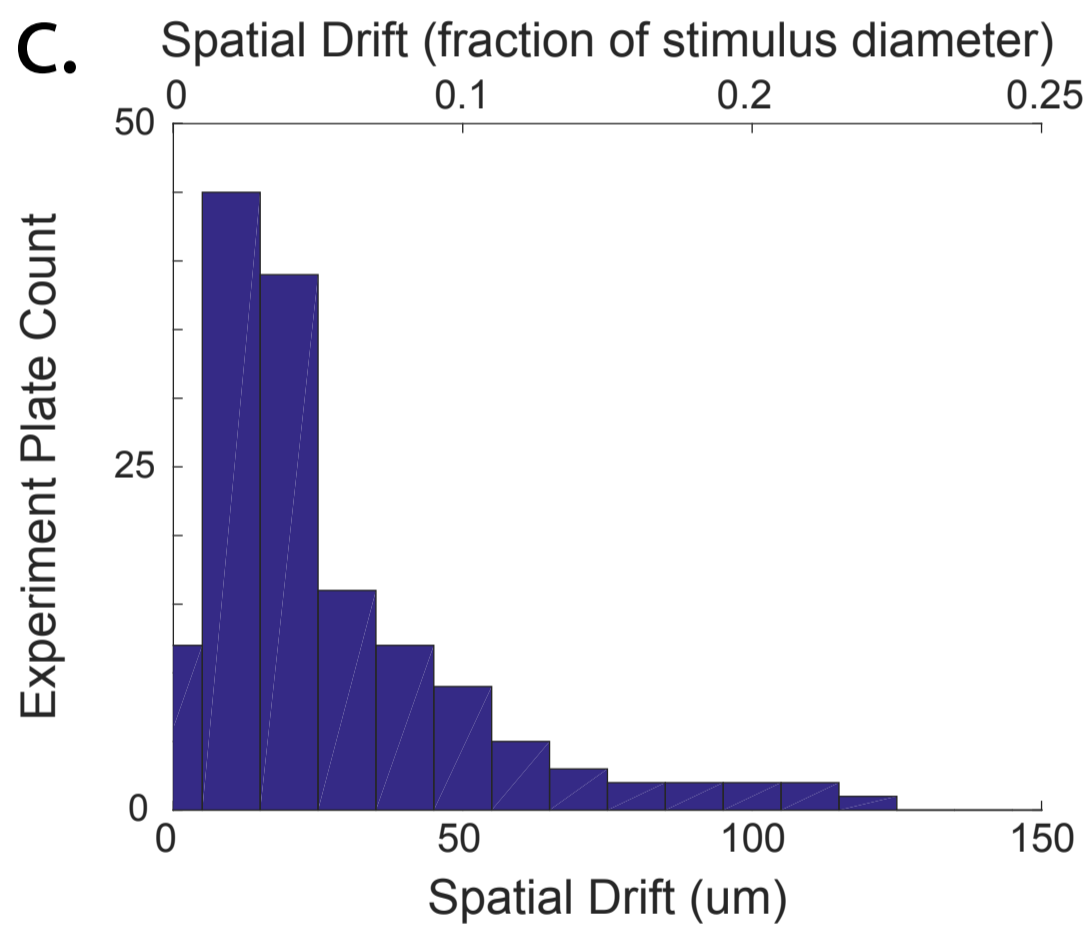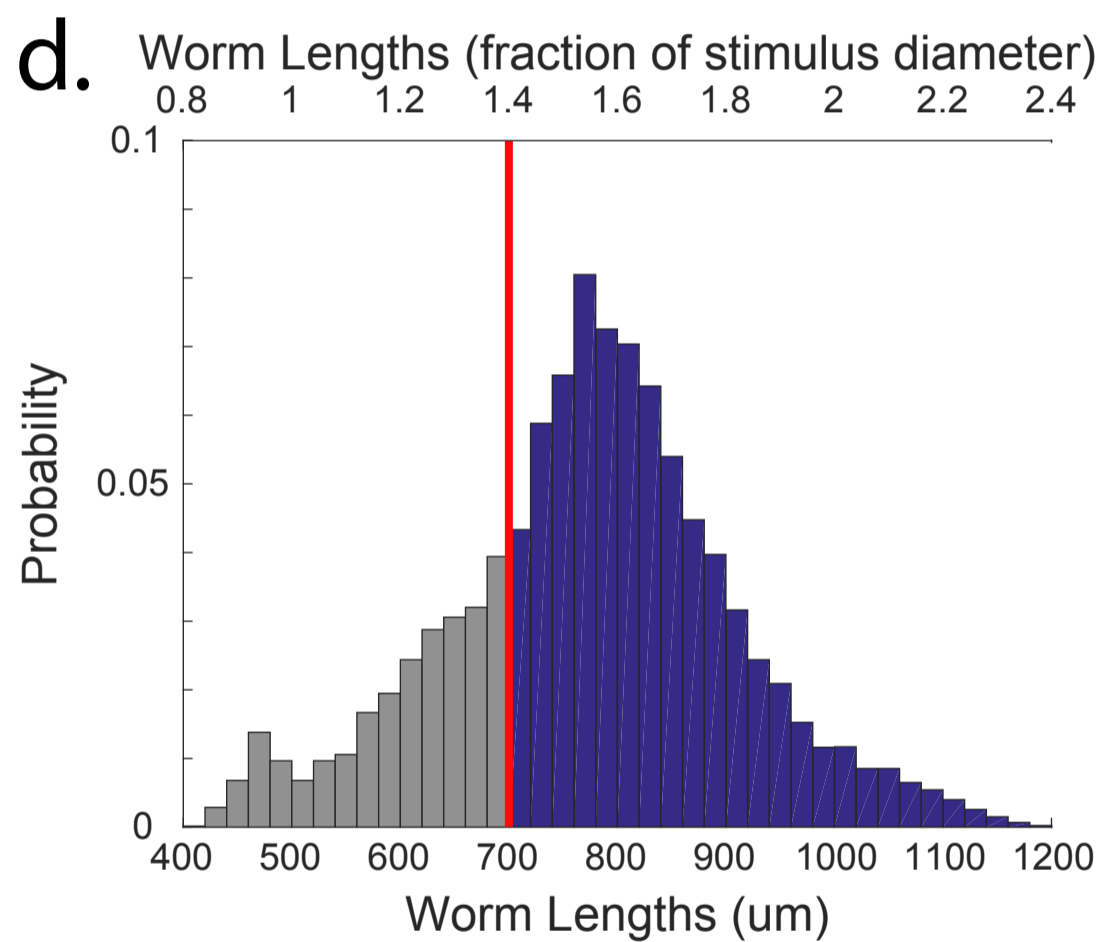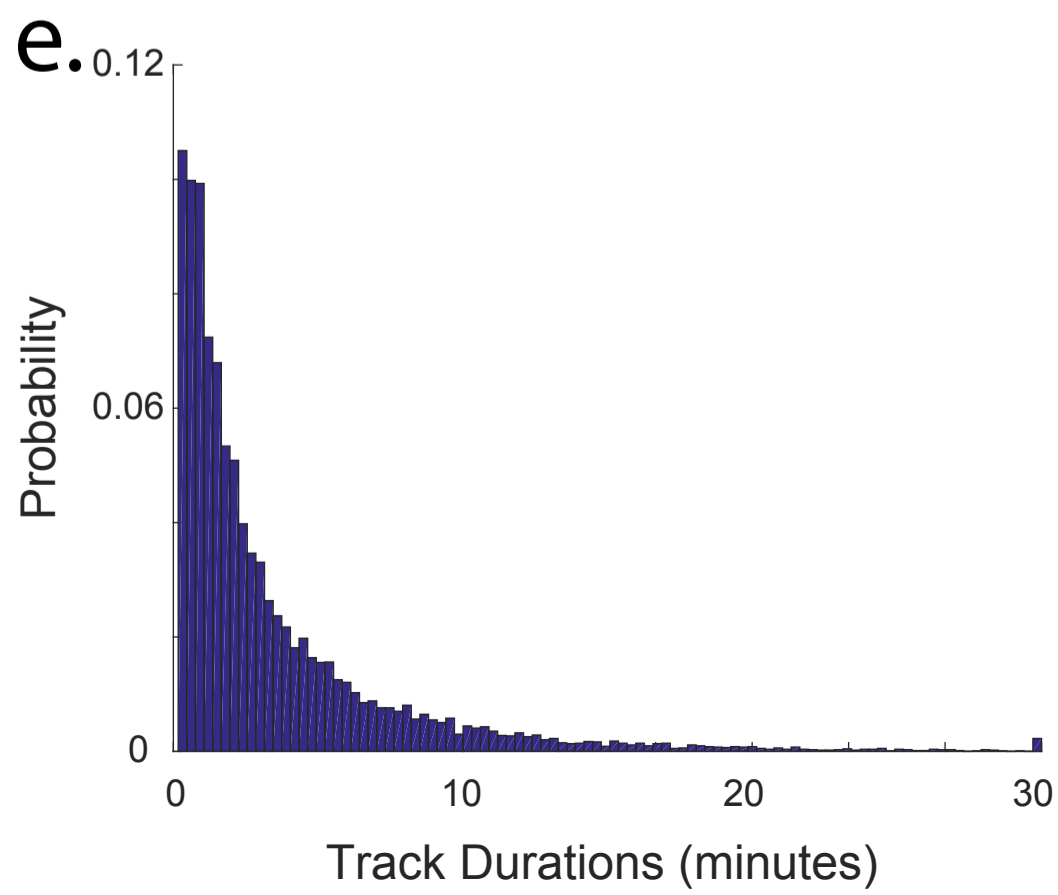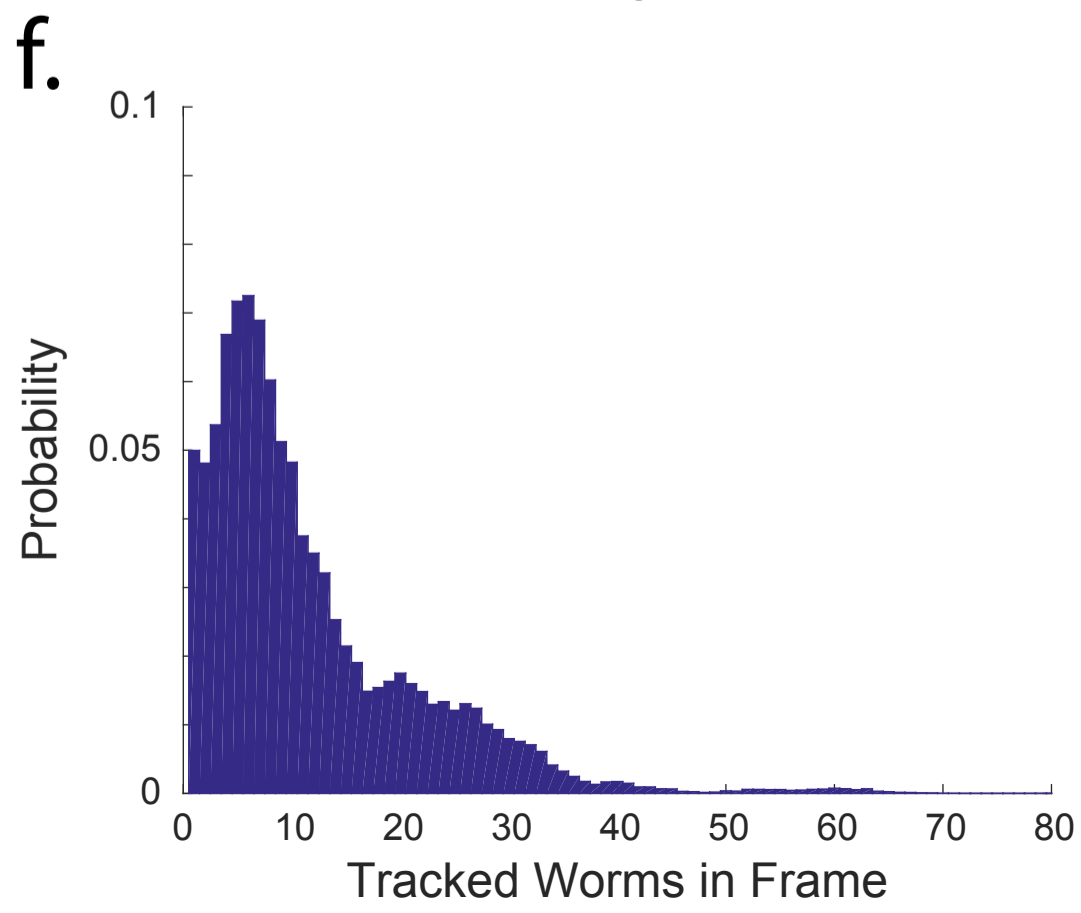

Supplement: S1 Fig — Performance is evaluated for the set of experiments related to anterior posterior mechanosensory stimluation, including those shown in Fig 3. (a) Acquire–draw–project latency is the latency associated with acquiring an image, drawing a new stimuli, and projecting that stimuli, as determined by visual time stamps drawn by the projector. (b) The probability distribution of update times for tracking worms is plotted on a log axis. Overall closed-loop latency is the sum of the acquire–draw–project latency and the tracking update time. (c) Histogram of camera-to-projector spatial drift between start and end of 30 minutes recording for each of 151 plates, as measured by a calibration pattern projected onto the agar. (d) Probability distribution of worm lengths for all tracks, as estimated by the extracted centerline of the worm. Only worms with lengths 700 um and above are included for behavioral analysis. (e) Probability distribution of the duration of tracks in the dataset. (f) The probability distribution of the number of tracked worms at any given time. The mean and standard deviation for this set of recordings is 12±10. Further breakdown of the mean animals per frame is listed in S1 Table. Machine-readable numerical values are listed in S5 Data. (PDF) [file pbio.3001524.s001.pdf]

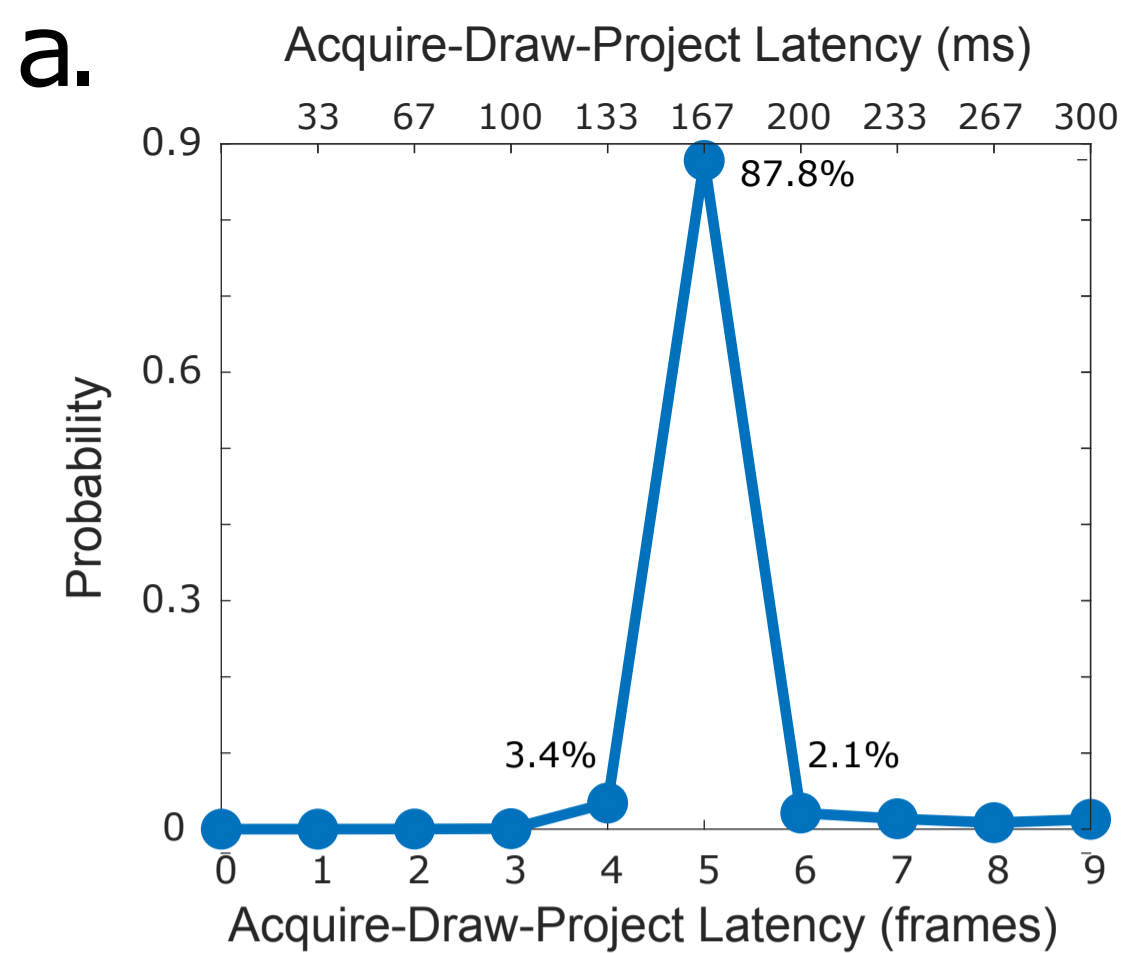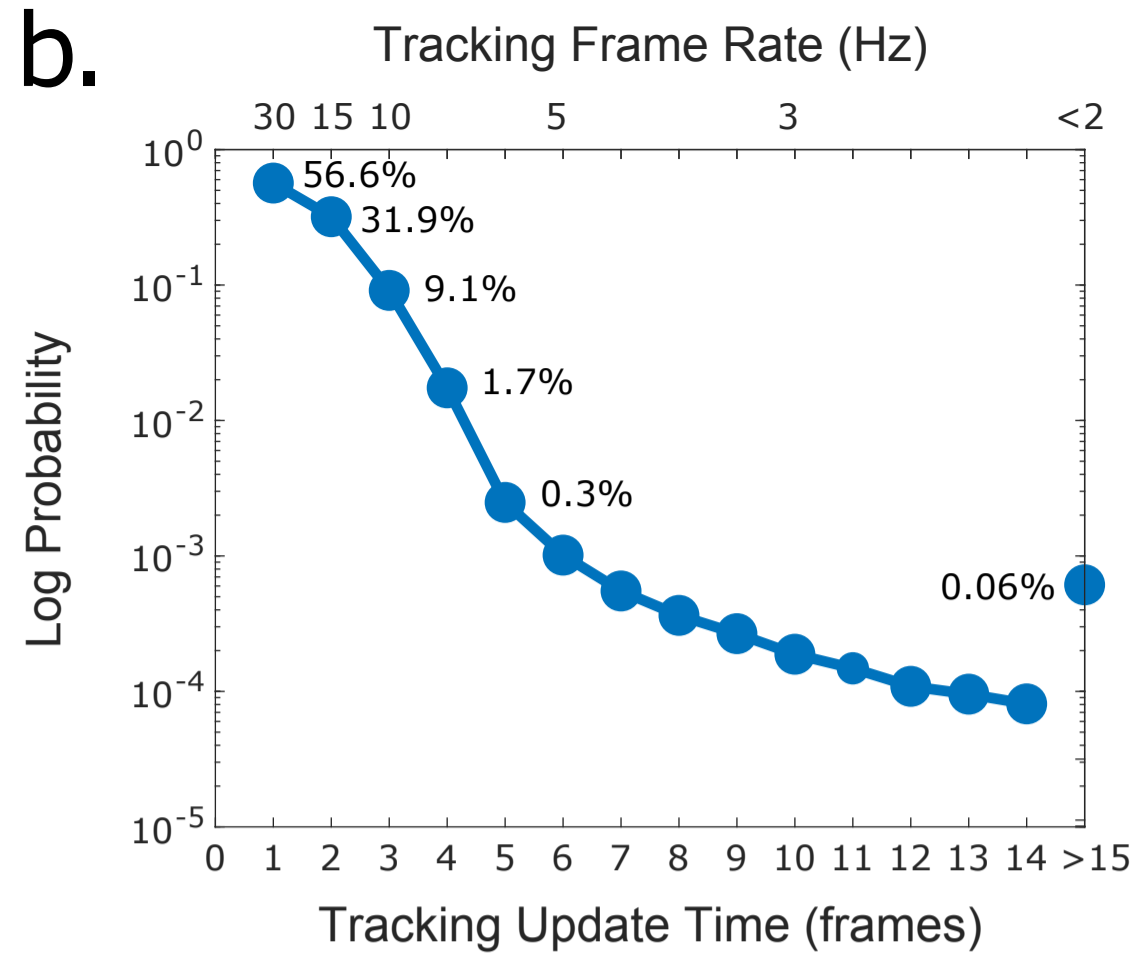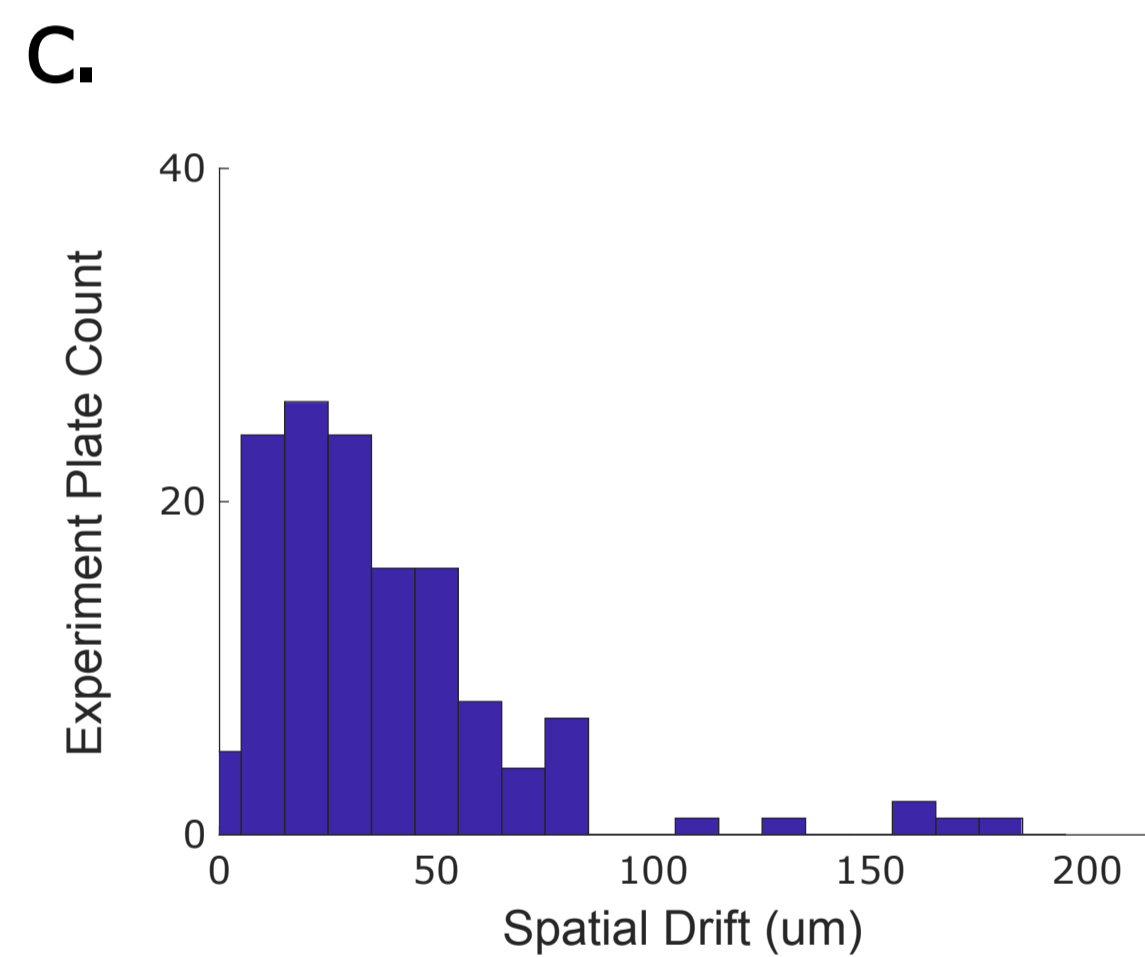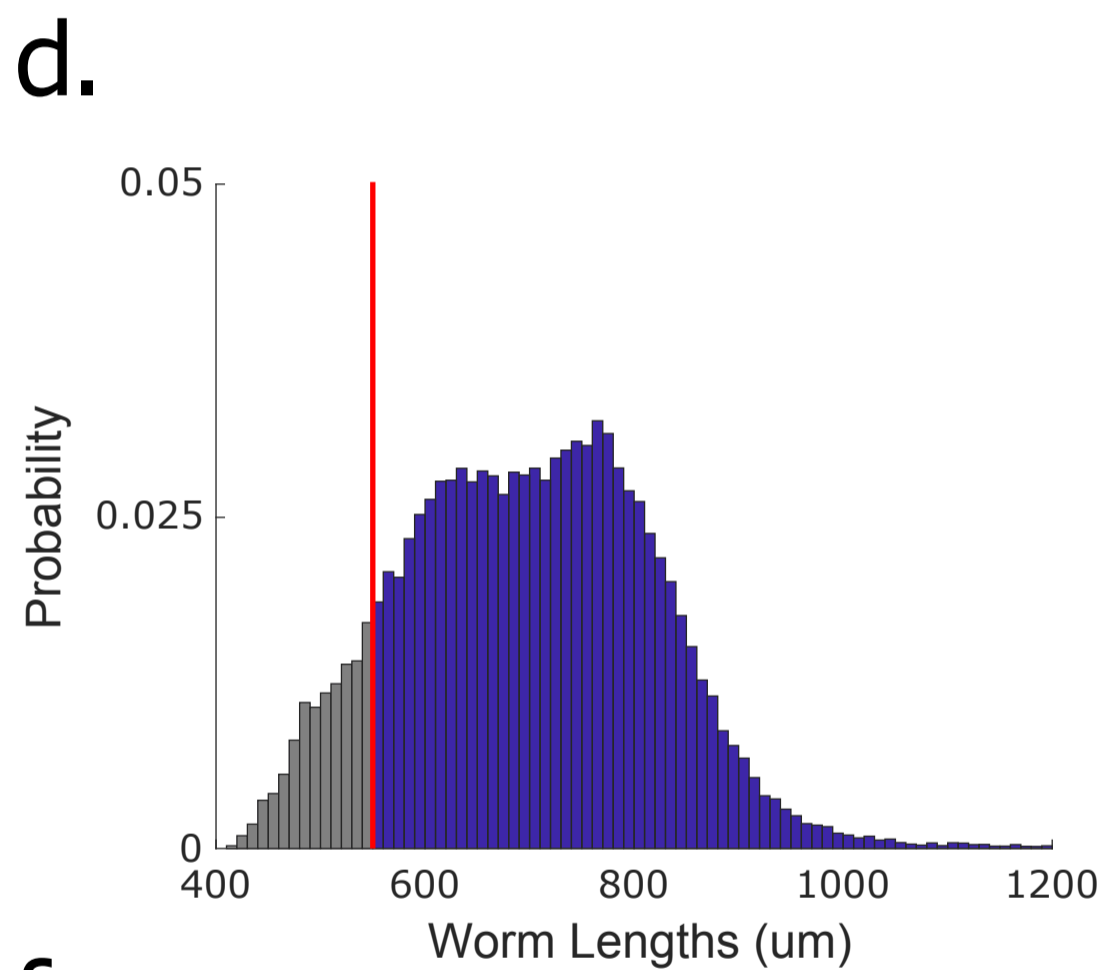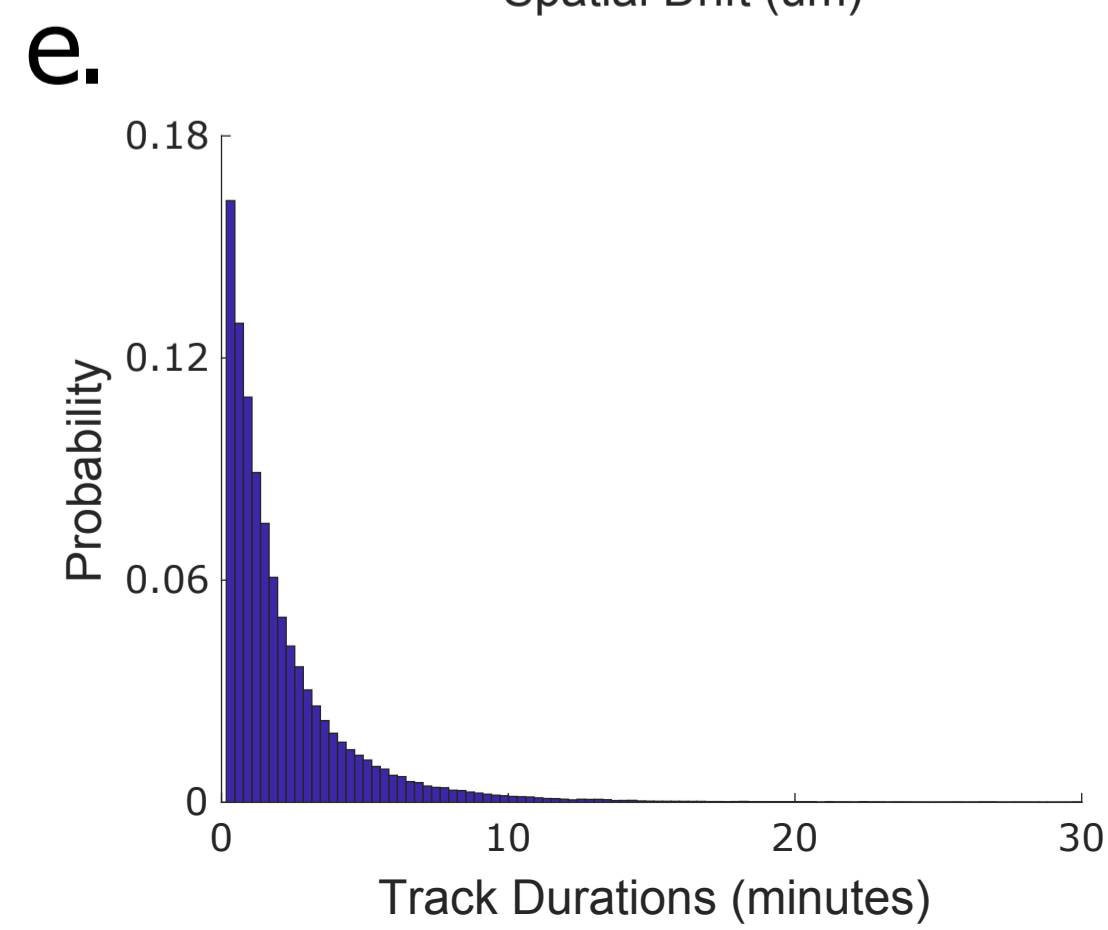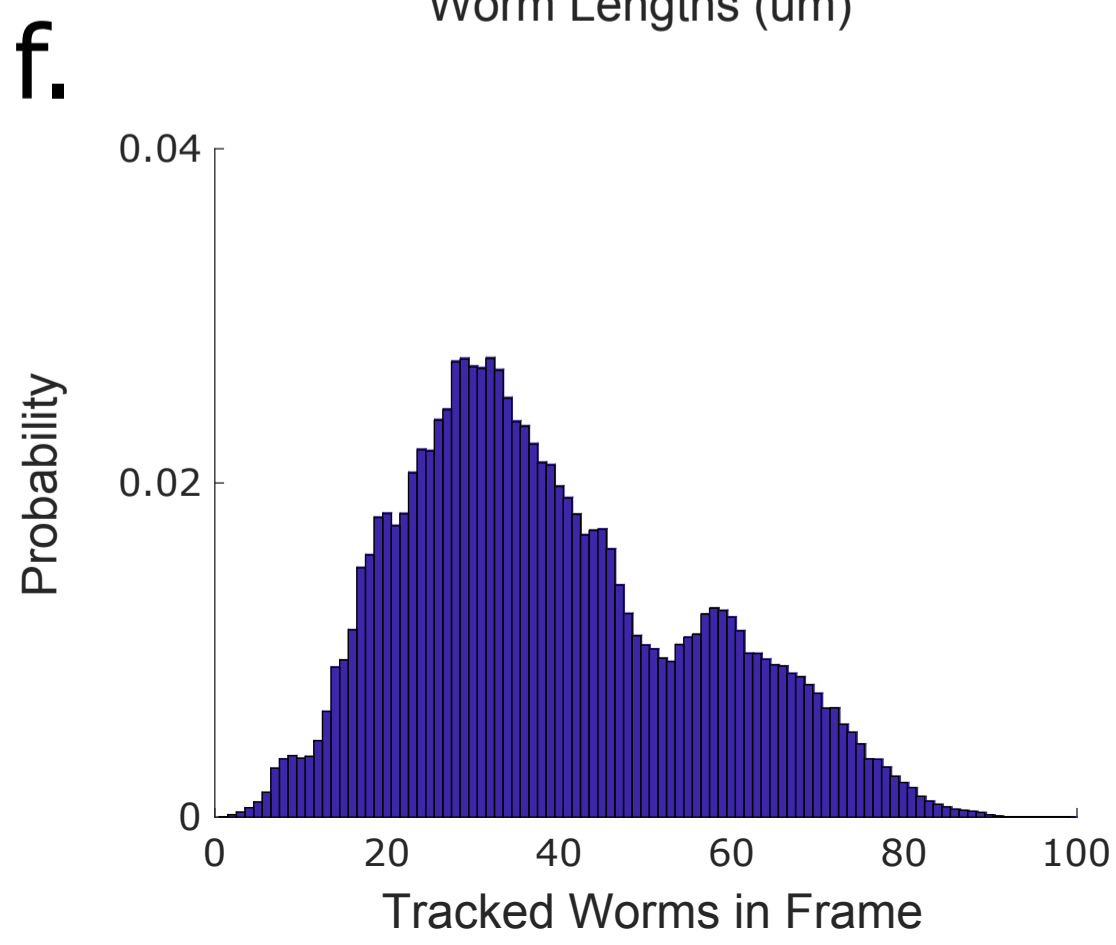

Supplement: S2 Fig — Performance is evaluated for the set of turning related experiments, including those in Fig 4, details of which are described in S1 Table. Subpanels are organized similarly to S1 Fig. For this set of recordings, 1-day-old animals were used, only worms with lengths 550 um and above were included in behavior analysis, and the mean number of tracked worms in a field of view per frame is 39. Machine-readable numerical values are listed in S6 Data. (PDF) [file pbio.3001524.s002.pdf]

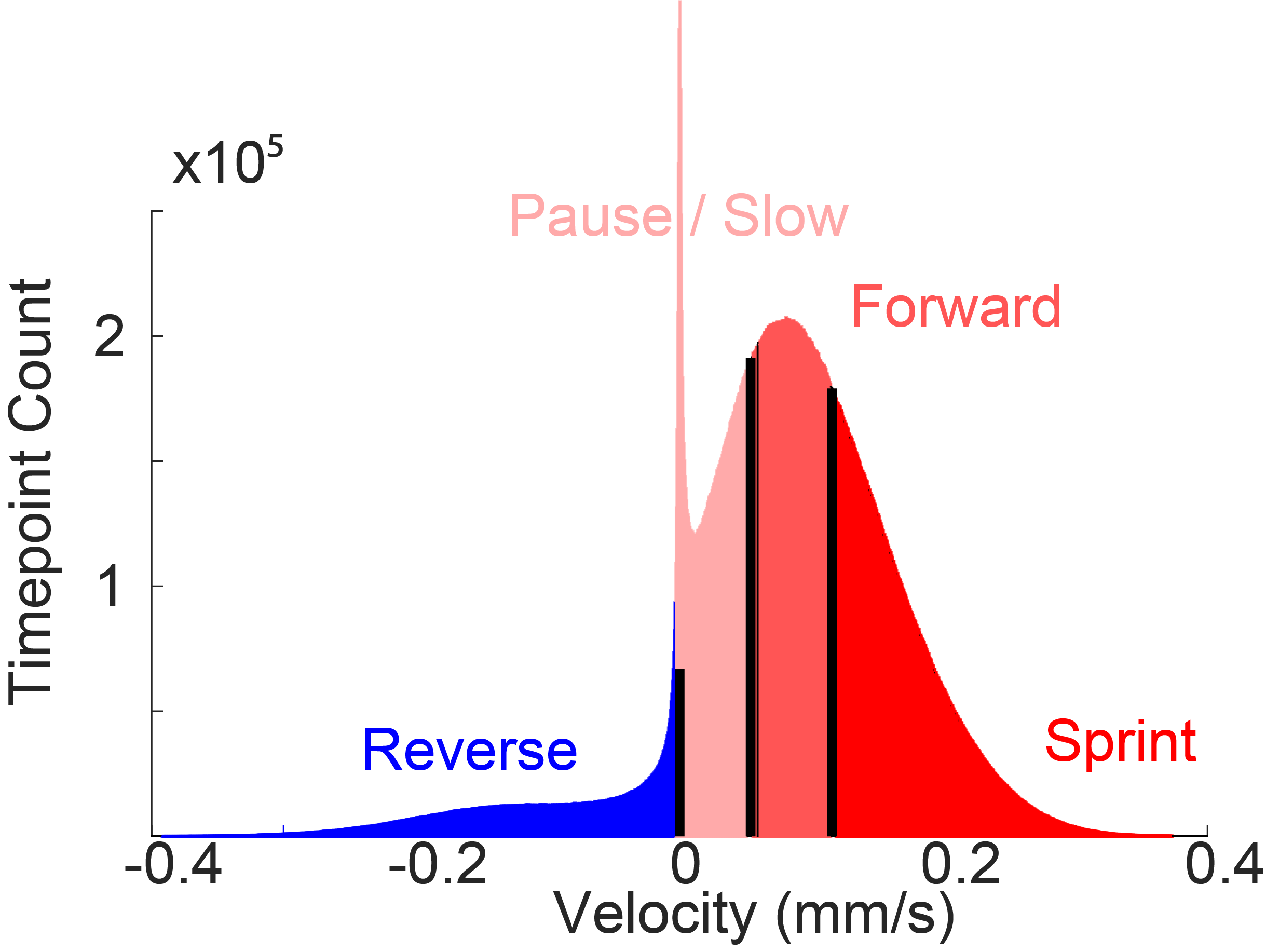

Supplement: S3 Fig — For the analyses performed in Figs 2 and 3, we classify animal behavior to be one of four states using the velocity distribution aggregated from both (+)ATR and (-)ATR head/tail stimulation experiments conducted with AML470. The “Reverse” state has a velocity of less than 0. The three states with positive velocity are divided so they are equally likely. The slowest state is “Pause/Slow.” The middle state is “Forward,”and the fastest state is “Sprint.” Machine-readable numerical values are listed in S7 Data. ATR, all-trans-retinal. (PNG) [file pbio.3001524.s003.png]

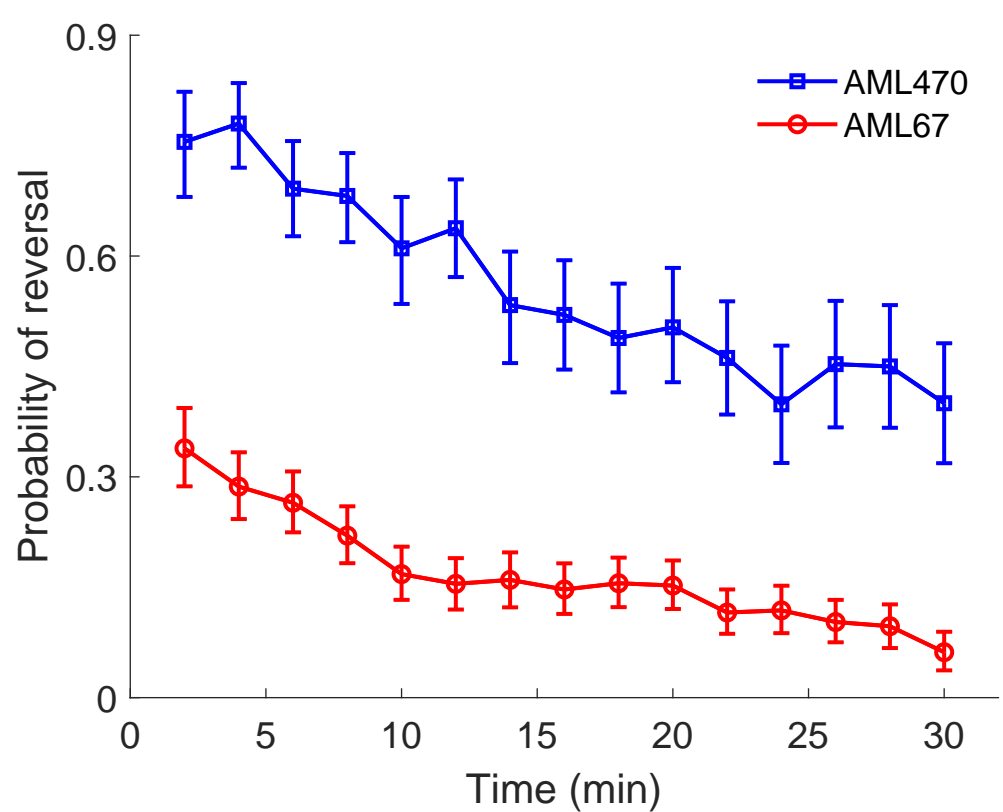

Supplement: S5 Fig — Open-loop optogenetic experiments are shown for strains AML67 (29 plates) and AML470 (20 plates) for only those stimuli that are 80 uW/mm2. Stimulation was delivered for 3 seconds at no more than once per 30 seconds. To aggregate responses across plates, stimulus events were binned into 2 minute intervals and the fraction of stimulus events resulting in reversals was reported. The error bars represent the 95% confidence interval calculated using 10,000 bootstraps. Machine-readable numerical values are listed in S9 Data. (PDF) [file pbio.3001524.s005.pdf]

## No Stimulus

## Tail Only

## Head Only

## Head and Tail

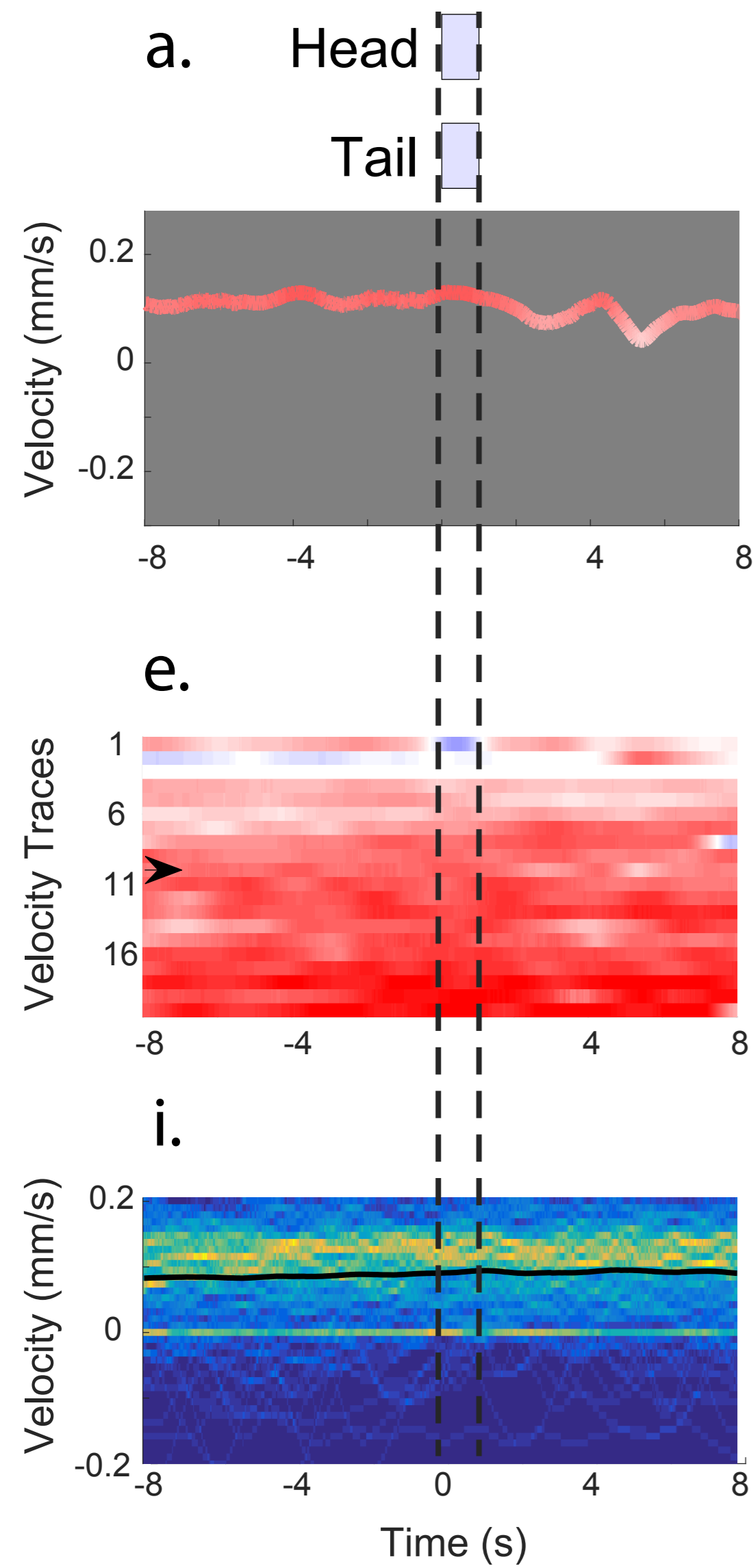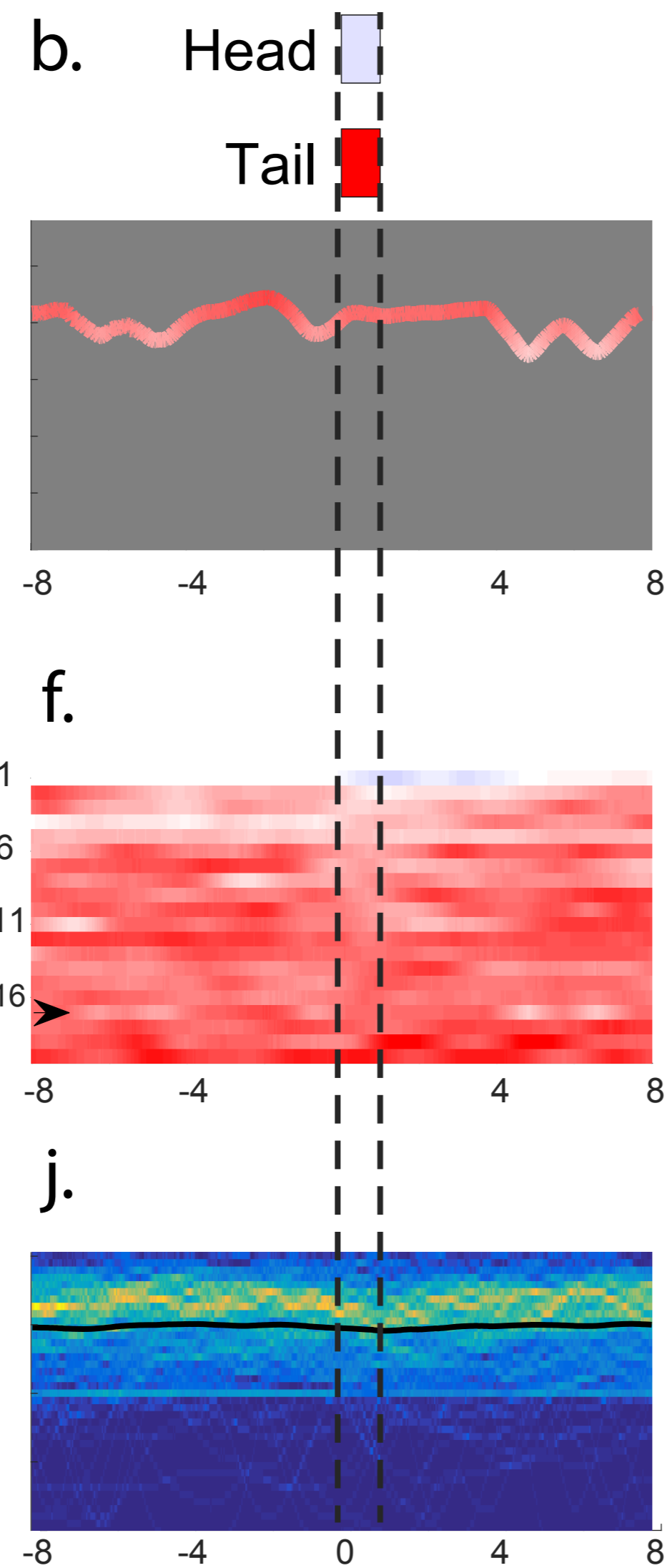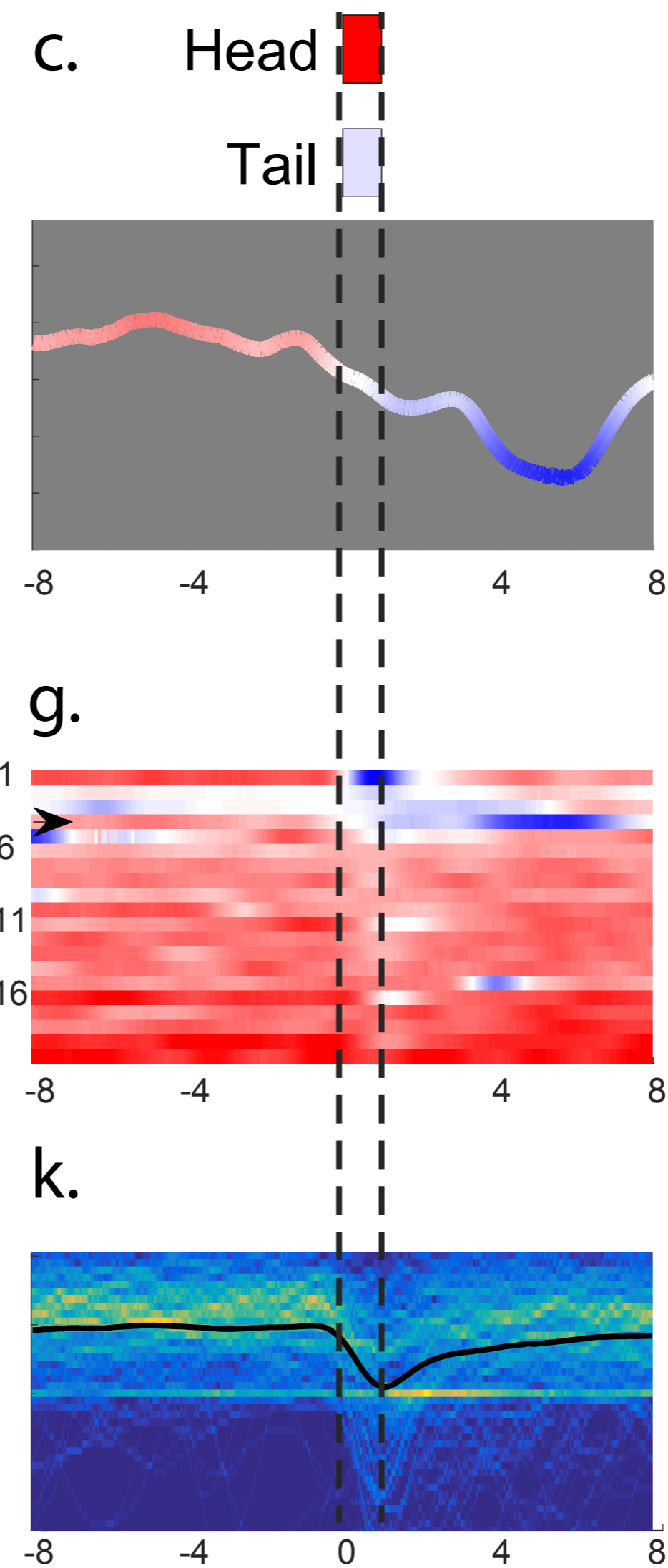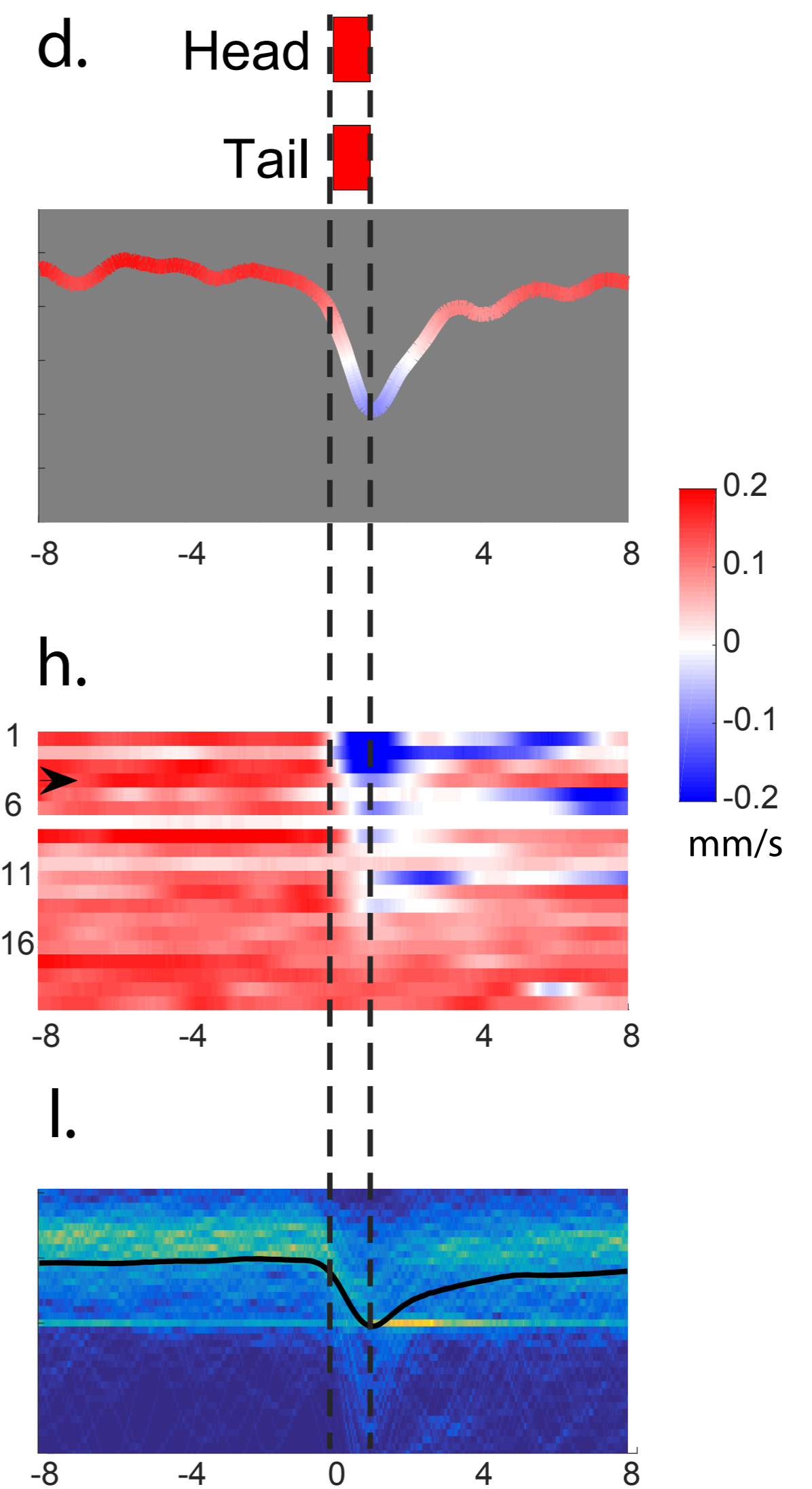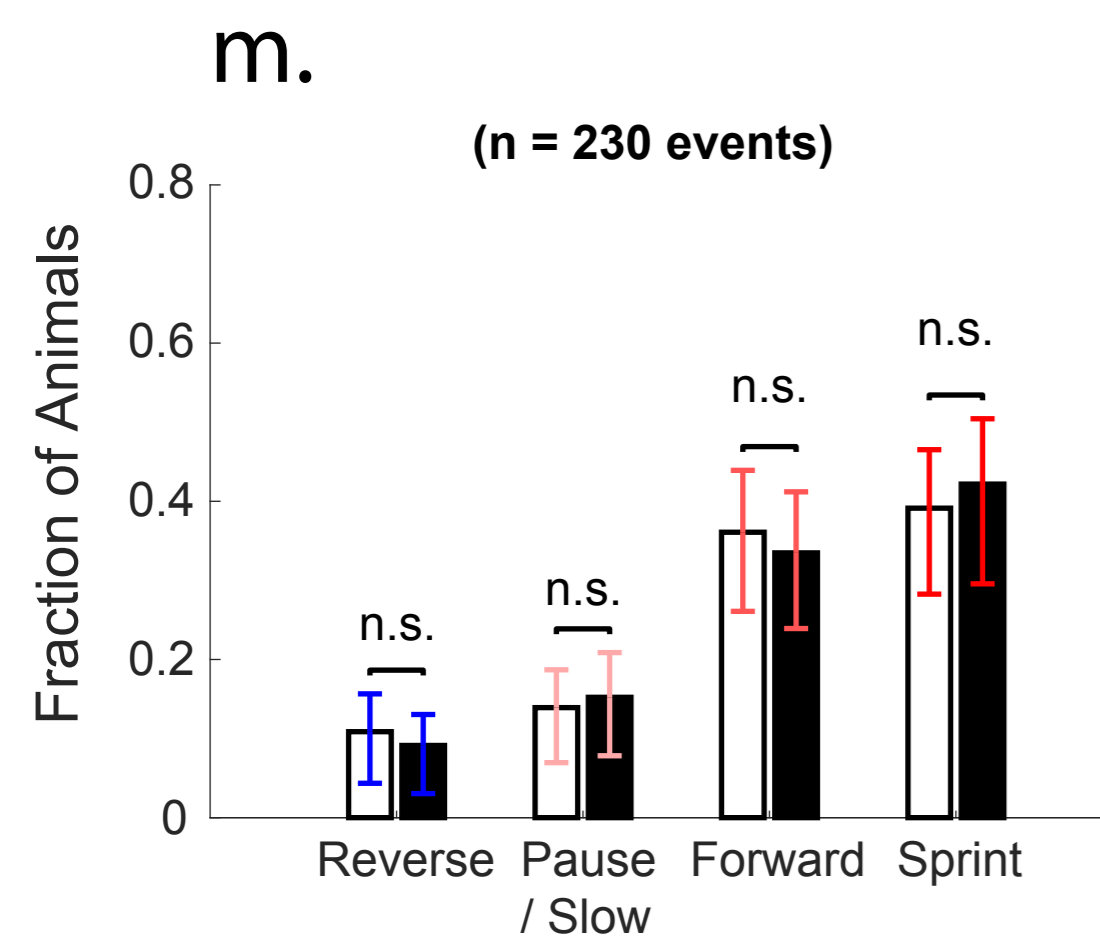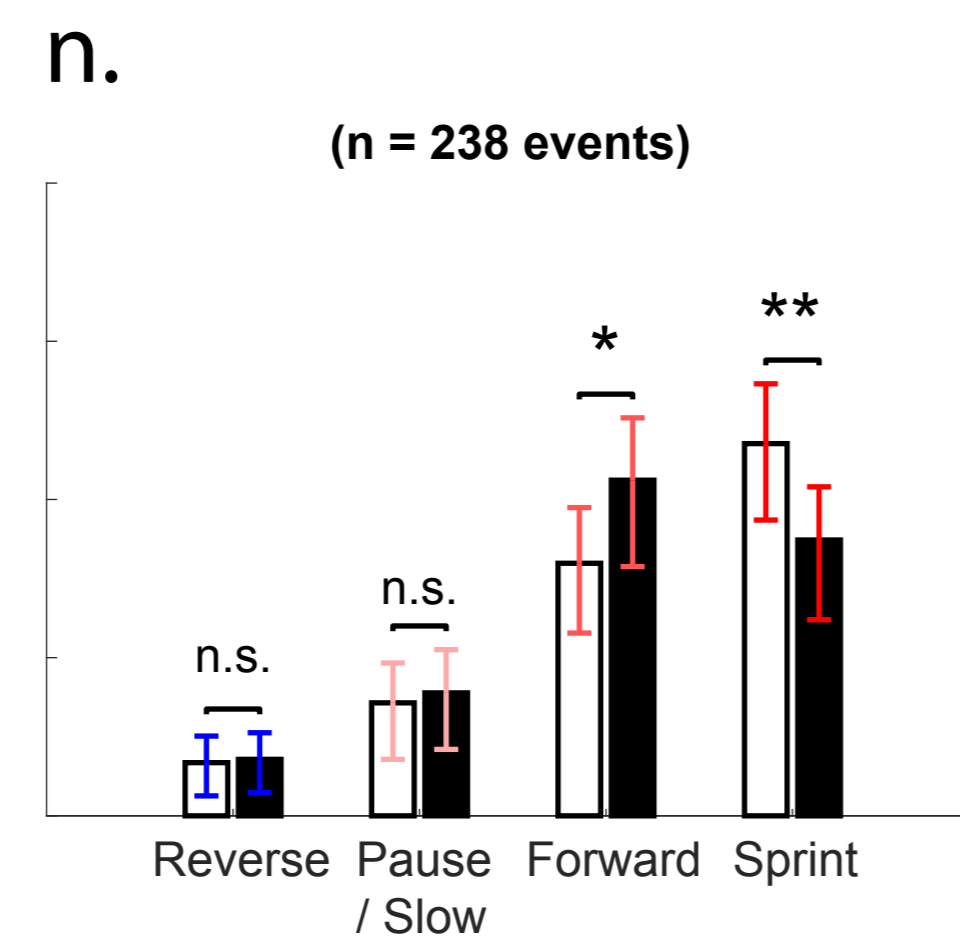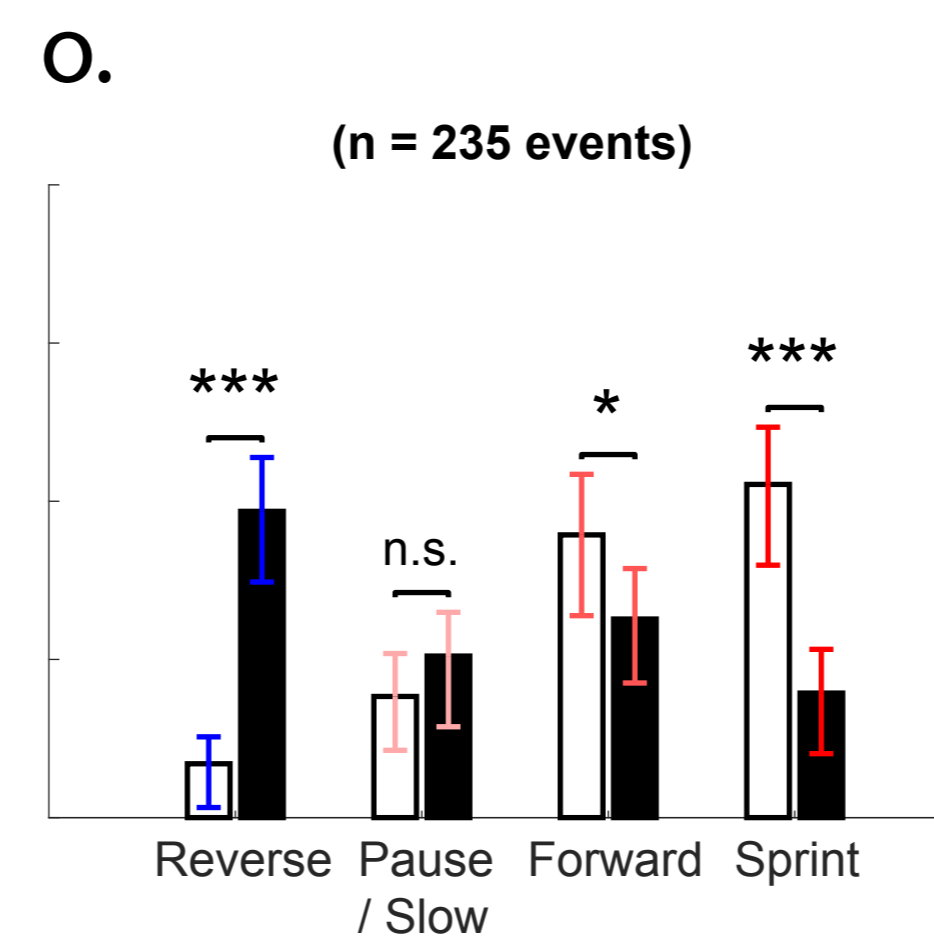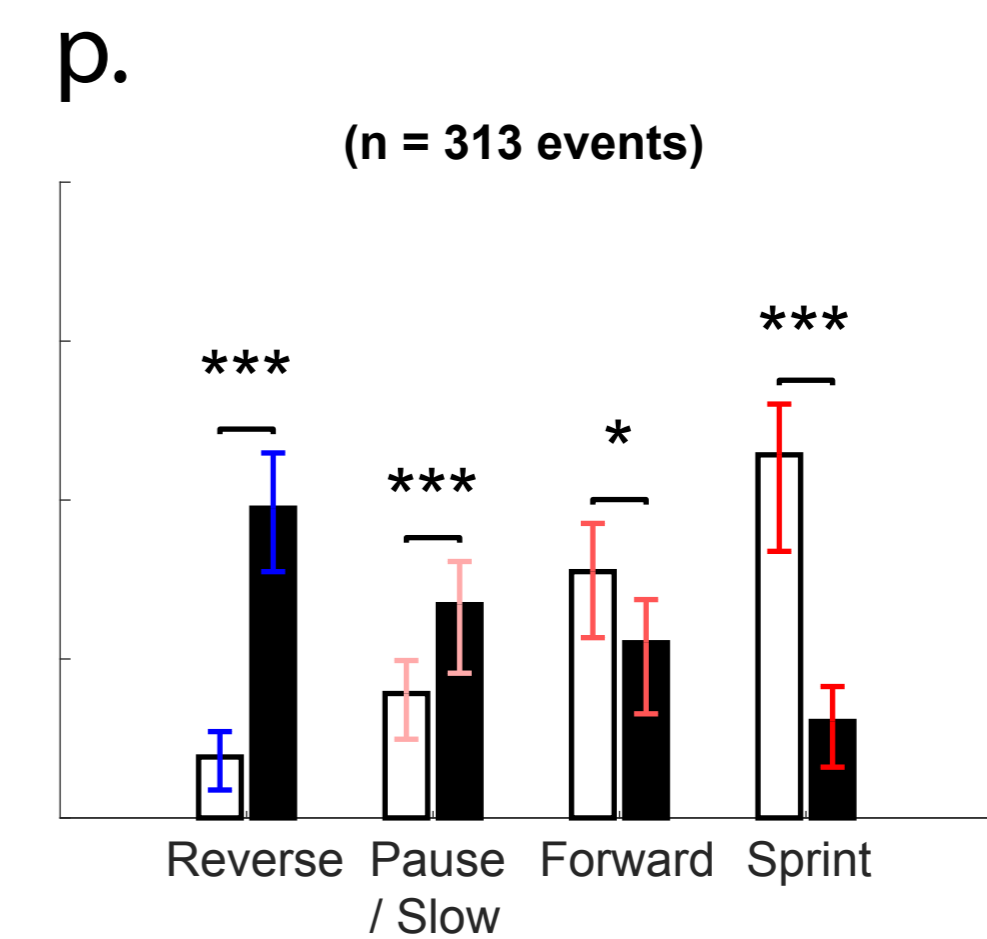

□ Before Stimulus ■ After Stimulus n.s.  $p > 0.05$  \*\*\*  $p < 0.001$  \*\*  $p < 0.01$  \*  $p < 0.05$

Supplement: S6 Fig — Anterior targeted stimuli evoke reversals but posterior stimuli unexpectedly reduce sprints for worms expressing Chrimson in the gentle touch mechanosensory neurons in this strain. Strain AML67 grown on retinal (+)ATR. (a–d) Select single animal velocity in response to no stimulation, tail only stimulation, head only stimulation, and combined head and tail stimulation respectively. Dotted lines denote the onset and termination of stimulation. The stimulus is a 1 second long 0.5-mm diameter circular dot centered at the tip of the animal’s head and/or tail with a red intensity of 80 uW/mm2 (0 uW/mm2 in “No Stimulus” condition)2. (e–h) Randomly selected velocity traces for 20 animals in each stimulus condition are color coded red/blue. The traces are sorted by the mean velocity during the 1-second stimulation window. The arrow indicates the selected velocity trace shown in the corresponding column in a–d. (i–l) Probability density of all tracked velocities for each stimulus condition. The mean velocity at any given time is overlaid as the black line. The number of stimuli events for this condition are shown below. (m–p) The fraction of animals belonging to behavioral states segmented by velocity before and after each stimulus condition. The before time point is taken 2 seconds prior to the stimulus onset, and the after time point is taken at the end of the 1-second stimulation. Significance between before and after is determined by p-values calculated using Wilcoxon rank sum test. Error bars represents 95% confidence intervals estimated using 1,000 bootstraps. Machine-readable numerical values are listed in S10 Data. (PDF) [file pbio.3001524.s006.pdf]
