# Supplementary material for: A high-throughput method to deliver targeted optogenetic stimulation to moving C. elegans populations
Source: PLoS Biol. 2022 Jan 28;20(1):e3001524. doi: 10.1371/journal.pbio.3001524 (PMC8827482; doi:10.1371/journal.pbio.3001524)
Supplement: S4 Fig — AML470 worms grown (-)ATR. The fraction of animals belonging to behavioral states segmented by velocity before and after each stimulus condition: (a) no stimulus, (b) tail only stimulus, (c) head only stimulus, and (d) combined head and tail stimulus. The before time point is taken 2 seconds prior to the stimulus onset, and the after time point is taken at the end of the 1-second stimulation. The stimulus is a 1 second long 0.5-mm diameter circular dot centered at the tip of the animal’s head and/or tail with a red intensity of 80 uW/mm2 (0 uW/mm2 in “No Stimulus” condition). Significance between before and after is determined by p-values calculated using Wilcoxon rank sum test. The significance test does not correct for multiple hypothesis testing. Error bars represents 95% confidence intervals estimated using 1,000 bootstraps. Machine-readable numerical values are listed in S8 Data. ATR, all-trans-retinal. (PDF) [file pbio.3001524.s004.pdf]

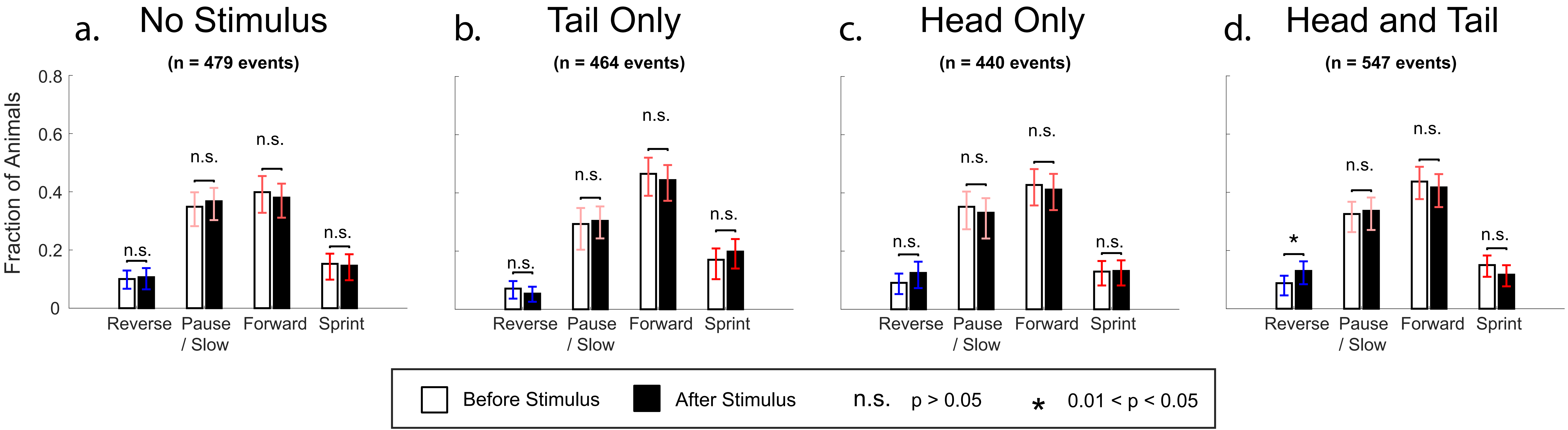
a.
No Stimulus
(n = 479 events)
b.
Tail Only
(n = 464 events)
c.
Head Only
(n = 440 events)
d.
Head and Tail
(n = 547 events)
Fraction of Animals
0.8
0.6
0.4
0.2
0
n.s.
*
Reverse
Pause / Slow
Forward
Sprint
Before Stimulus
After Stimulus
n.s. p > 0.05
* 0.01 < p < 0.05
