# Supplementary material for: A high-throughput method to deliver targeted optogenetic stimulation to moving C. elegans populations
Source: PLoS Biol. 2022 Jan 28;20(1):e3001524. doi: 10.1371/journal.pbio.3001524 (PMC8827482; doi:10.1371/journal.pbio.3001524)
Supplement: S1 Table — “Stim events on valid animals” refers to those stimulation events that landed on animals that met our inclusion criteria for a worm, including criteria for worm shape, size, and track duration, and minimum movement. Note that here stimulation events for all stimulation intensities and durations are listed, whereas in many of the figures only a single stimulation intensity duration is reported. (PDF) [file pbio.3001524.s007.pdf]

Experiment Series	Type	Stim Duration (s)	Illumination Intensity (uW/mm2)	Illumination Diameter (mm)	ISI (s)	Strain	ATR	Plates	Days	Total stim events	Cumulative Recording Length (animal-hours)	Animals per frame (Mean ± Stdev)	Stim events on valid animals	Figures
Anterior vs Posterior Stimulation	Open Loop	1	0, 20, 40, 60, 80	0.5	30	AML470	+	95	4	74,693	539	13 ± 10	43,418	Figures 1,2,3; Supplementary Figures S1, S3
							-	28		19,293	144	12 ± 10	11,744	Supplementary Figures S1, S3, S4
						AML67	+	24	1	10,911	80	8 ± 6	7,001	Supplementary Figure S6
							-	4		567	3.6	6 ± 5	284	-
Stimulation during turning vs forward	Open-loop	1,3,5	0.5, 40, 80	1.5	30	AML67	+	29	3	73,775	633	42 ± 18	39,769	Figure 4; Table 1; Supplementary Figure S5
	Closed-loop triggered on turns				>30		+	47		40,600	1060	39 ± 22	9,776	Figure 4; Table 1
	Open-loop	3	0.5, 20, 40, 60, 80		30	AML470	+	20		38,581	296.8	36 ± 12	13,132	Figure 4; Supplementary Figure S5
	Closed-loop triggered on turns				>30		+	55		58,382	945.6	38 ± 12	7,550	Figure 4
