# Supplementary material for: A high-throughput method to deliver targeted optogenetic stimulation to moving C. elegans populations
Source: PLoS Biol. 2022 Jan 28;20(1):e3001524. doi: 10.1371/journal.pbio.3001524 (PMC8827482; doi:10.1371/journal.pbio.3001524)
Supplement: S2 Table — Hardware parts list for the instrument. The frame is made from 1 inch aluminum extrusions and are not included. The unit count is for each instrument. (PDF) [file pbio.3001524.s008.pdf]

Manufacturer	Item	Model/Parts No.	Units	Notes
Camera				
Basler	Camera	acA4112-30um	1	
Basler	USB Cable	Basler 2000035994	1	
Basler	$\frac{1}{4}$-20 Mounting Adapter<br>for Basler ace L USB 3.0	Basler 2200000191	1	
Basler	Digital I/O and power cable,<br>6-pin Hirose (Female), 10 m	Basler 2000029411	1	
Lens And Filter				
Fujinon	CF16ZA-1S 16mm f/1.80<br>Machine Vision C-Mount Lens	CF16ZA-1S	1	
Edmund Optics	M37.5 x 0.5 Empty Filter Mount	67-684	1	
Thorlabs	C-Mount Spacer Ring, 2.00 mm Thick	CMSP200	1	
Semrock	538/40 nm BrightLine single-band<br>bandpass filter, 35mm unmounted	FF01-538/40-35-D	1	Also allows<br>in IR >800nm
SCHOTT	OG-550, 50.8mm Sq., Longpass Filter	OG-550	1	
Roscolux	3" x 5" 200 filters, Color Filter<br>Booklet, #318Mayan Sun	39-418	1	35 mm circles<br>are cut out.<br>2 filters are<br>used per lens.
Projector				
Anhua	M5NP Structured light<br>projector engine for 3D Measuring	M5NP	1	
Wintech	DLP4500 Projection DLP Reference<br>Design Evaluation Board<br>without optical module	LCR4500 VIS KIT	1	
Kaga Electronics USA	AC/DC DESKTOP ADAPTER 12V 84W	KTPS90-1207	1	
Tripp Lite	6' USB A TO MINI-B CABLE M/M	UR030-006	1	
Tripp Lite	HDMI TO MINI HDMI CABLE 6'	P571-006-MINI	1	
CNC Tech	CORD 18AWG 5-15P -<br>320-C5 6' BLK	800-18-39B-BL-0006F	1	
Molex	Socket Contact Tin<br>24-26 AWG Crimp Stamped	874210000	50	
Molex	9 Position Rectangular Housing<br>Connector Receptacle Natural<br>0.059" (1.50mm) for evaluation board	87439-0900	1	
Molex	6 Position Rectangular Housing<br>Connector Receptacle Natural<br>0.059" (1.50mm) for evalutaion board	87439-0600	3	
Molex	Fan connector	51021-0300	1	
Molex	Fan and optical module terminal crimp	50079-8100	20	

Molex	6 Position Rectangular Housing Connector Receptacle Natural 0.049” (1.25mm) Optical Module LED	51021-0600	3	
Foxconn	Fan	PVA030E12M	1	

IR Ring				
LightingWill	DC12V 5M/16.4ft 72W SMD5050 300LEDs IR InfraRed 850nm Tri-chip White PCB Flexible LED Strips 60LEDs 14.4W/M	B01DM9BL5I	1	
DROK	DC Car Power Supply Voltage Regulator Buck Converter 8A/100W 12A Max DC 5-40V to 1.2-36V Step Down Volt Convert Module	90483	1	
CENTROPOWER	10 Pairs DC Power Pigtail Cable 12V 5A Male Female Connectors DC Cable for CCTV Security Camera Power Adapter Connectors	CT-DCCORD	1	
Marshall	Potentiometer - 22K Linear, Marshall, 16mm	”0609722158497”	1	
TDK-Lambda Americas	AC/DC CONVERTER 24V 27A 600W	285-1742-ND	1	
LE PAON	Embroidery Hoops Plastic Cross Stitch Hoop 7 Pcs	4336933944	1	Only the 6 in hoops were used
Thorlabs	Black Hardboard, 24” x 24” (610 mm x 610 mm), 3/16” (4.76 mm) Thick, 3 Sheets	TB4	1	

Computer				
Origin PC	Custom Rackmount PC with AMD 3970X Processor	L-Class	1	
Sabrant	2TB Rocket NVMe PCIe M.2 2280 Internal SSD High Performance Solid State Drive	SB-ROCKET-2TB	4	
